# The Therapeutic Landscape of Nausea and Vomiting of Pregnancy and Hyperemesis Gravidarum: Five Decades of Evolving Treatment and Supportive Care

**DOI:** 10.3390/medsci14030419

**Published:** 2026-07-22

**Authors:** Flavia Gabriela Ban, Andrei-Flavius Radu, Ada Radu, Delia Mirela Tit, Gabriela S. Bungau

**Affiliations:** 1 Doctoral School of Biological and Biomedical Sciences, University of Oradea, 410087 Oradea, Romania; blaga.flaviagabriela@student.uoradea.ro (F.G.B.); dtit@uoradea.ro (D.M.T.); gbungau@uoradea.ro (G.S.B.); 2 Department of Psycho-Neuroscience and Recovery, Faculty of Medicine and Pharmacy, University of Oradea, 410073 Oradea, Romania; 3 Department of Pharmacy, Faculty of Medicine and Pharmacy, University of Oradea, 410028 Oradea, Romania

**Keywords:** nausea and vomiting of pregnancy, hyperemesis gravidarum, bibliometric analysis, PELT, science mapping, Web of Science

## Abstract

**Background/Objectives**: Among the most common medical conditions of pregnancy, nausea and vomiting of pregnancy (NVP) and its severe form hyperemesis gravidarum (HG) represent a clinically significant and increasingly researched area of maternal health. However, the global evolution, structural organization, and thematic development of this literature remain insufficiently characterized. This bibliometric study maps five decades of global research on therapeutic and supportive interventions for NVP and HG. **Methods**: Web of Science Core Collection records were searched on 10 May 2026, restricted to English-language articles and reviews published up to 2025, and deduplicated to 1182 unique documents. A secondary thematic filter was subsequently applied to generate a refined obstetric subset for the country collaboration, keyword co-occurrence, and thematic evolution analyses. Bibliometric performance, collaboration networks, source impact, institutional output, PELT-based changepoint detection, metadata completeness, keyword co-occurrence, and thematic evolution were analyzed using VOSviewer, Bibliometrix/Biblioshiny, and custom Python scripts. **Results**: Publication output increased markedly after 2010 and reached its maximum in 2025. PELT sensitivity analysis localized the principal transitions to the mid-1990s and early 2010s, informing three broad analytical macroperiods: 1975–1994, 1995–2009, and 2010–2025. The United States dominated cumulative output and collaboration intensity, while Canada, England, Australia, Italy, Germany, and China showed substantial scientific influence. Core publication venues were concentrated in obstetrics, gynecology, reproductive safety, and pregnancy-focused clinical medicine. Keyword and thematic analyses showed a transition from early vomiting, Bendectin, exposure, and pregnancy-safety concerns toward pharmacological treatment, complementary interventions, controlled clinical evaluation, maternal–fetal outcomes, and supportive management. Metadata-completeness analysis identified a major pre-1991 indexing discontinuity, requiring caution when interpreting long-range keyword-based trends. **Conclusions**: These findings outline the trajectory of the field and highlight priorities for future research, including stronger comparative evidence for pharmacological, complementary, and supportive-care strategies, broader international collaboration, and prospective studies to strengthen the evidence base for managing NVP and HG.

## 1. Introduction

Nausea and vomiting in pregnancy (NVP) affect approximately 50–80% of pregnancies and range from self-limited symptoms to hyperemesis gravidarum (HG), a severe condition associated with dehydration, electrolyte disturbances, nutritional impairment, and hospitalization. Beyond physical manifestations, severe disease may substantially impair maternal quality of life, psychosocial functioning, sleep, and daily activities, while also contributing to adverse obstetric outcomes [1,2,3].

Current evidence supports a multifactorial pathophysiology involving hormonal, placental, genetic, gastrointestinal, metabolic, and psychosocial mechanisms. Recent mechanistic studies have increasingly implicated the GDF15–GFRAL signaling pathway in disease susceptibility and severity, linking placental signaling with maternal sensitivity to emetic stimuli [4,5].

Clinical presentation and therapeutic requirements vary considerably across the disease spectrum, prompting individualized and severity-based management strategies. Contemporary care increasingly incorporates patient-centered functional assessment alongside biochemical evaluation, while emphasizing objective symptom stratification, outpatient-oriented supportive care, and avoidance of both undertreatment and unnecessary pharmacological escalation [6,7].

Despite the broad range of therapeutic approaches investigated for NVP and HG, including antihistamines, dopamine antagonists, serotonin receptor antagonists, corticosteroids, intravenous hydration, nutritional support, and complementary interventions, clinical evidence remains heterogeneous across disease severities and study designs. Variability in outcome measures, comparators, and treatment protocols continues to limit the establishment of consistently standardized management strategies [8].

Beyond conventional pharmacological management, complementary and integrative approaches such as acupuncture are increasingly explored, particularly among patients concerned about fetal drug exposure. However, recent consensus-based evidence highlights persistent variability in therapeutic protocols, adjunctive interventions, and supportive care frameworks across clinical settings [9].

Bibliometric and science-mapping methodologies are increasingly used in obstetrics and gynecology to evaluate publication dynamics, thematic evolution, and collaborative research structures across rapidly expanding clinical fields such as preeclampsia and endometriosis. Approaches integrating co-authorship analysis, keyword clustering, and longitudinal thematic mapping have proven useful for identifying emerging research directions and structural patterns that are difficult to capture through conventional evidence synthesis alone [10,11].

In the field of NVP and HG, research activity has expanded considerably in recent decades. However, the literature remains affected by heterogeneous diagnostic definitions, inconsistent outcome reporting, and fragmented therapeutic evidence. Variability in symptom severity assessment, hospitalization criteria, and treatment-related outcome measures continues to limit comparability across studies and complicates evidence synthesis. Although initiatives such as the Windsor definition and core outcome standardization efforts have sought to improve research harmonization, implementation across published studies remains incomplete [12,13,14].

Despite the increasing scientific interest in pregnancy-related nausea, vomiting, and HG, no dedicated bibliometric investigation has comprehensively evaluated this field as an integrated research domain. Existing evidence syntheses have primarily focused on clinical efficacy, management strategies, or priority research questions, while publication trends, thematic development, institutional productivity, and international collaboration patterns remain insufficiently characterized. This gap highlights the need for a structured longitudinal science-mapping analysis capable of providing a broader perspective on the evolution and organization of research within this field [13,14].

Given the growing but methodologically heterogeneous body of literature on pregnancy-related nausea, vomiting, and HG, this study aimed to provide, to the best of our knowledge, the first comprehensive bibliometric and science-mapping analysis dedicated exclusively to this research domain. Specifically, the study sought to characterize longitudinal publication trends, thematic evolution, institutional productivity, source impact, and international collaboration patterns related to therapeutic and supportive interventions for NVP and HG, thereby addressing the current lack of an integrated large-scale overview of the field’s scientific development and research structure.

## 2. Materials and Methods

### 2.1. Data Source and Search Strategy

The present bibliometric study was conducted using the Web of Science Core Collection as the primary data source. This database was selected because it provides structured bibliographic records, standardized citation data, and broad coverage of peer-reviewed scientific literature across biomedical, pharmacological, obstetric, and public health domains. The use of a single comprehensive database also allowed methodological consistency and avoided the difficulties associated with merging records from multiple databases, such as duplicate entries, divergent citation formats, and differences in journal indexing.

The search encompassed all available WoSCC sub-indexes, including the Science Citation Index Expanded, Social Sciences Citation Index, Arts & Humanities Citation Index, Conference Proceedings Citation Indexes (Science and Social Science & Humanities), Book Citation Indexes, Emerging Sources Citation Index, Current Chemical Reactions, and Index Chemicus. Although several of these sub-indexes fall outside the core scope of NVP and HG research, they were retained during the primary query to ensure transparent reporting of the complete WoSCC retrieval framework. To maintain scientific relevance and methodological rigor, the subsequent dataset was restricted exclusively to peer-reviewed articles and review articles.

The search was performed on 10 May 2026, and included documents published up to and including 2025. A fixed end-year was deliberately chosen to create a reproducible dataset, as open-ended searches fluctuate daily as newly indexed records are added. For example, due to inherent database indexing lags, an attempt to replicate a search including the ongoing year of 2026 would yield entirely different publication volumes if conducted in May versus August, as backlogged spring publications finally enter the database. Excluding the incomplete calendar year prevents this temporal distortion and preserves the reliability of the longitudinal trend analyses.

The search strategy was developed to capture three major conceptual domains: the clinical condition itself, including NVP, HG, morning sickness, emesis gravidarum, and related pregnancy-specific terminology; pharmacological, non-pharmacological, nutritional, and supportive interventions used in the management of NVP and HG; and specific commercial or historical names of doxylamine–pyridoxine formulations. The search was conducted using the Topic field tag, TS, which retrieves terms from titles, abstracts, author keywords, and Keywords Plus.

The primary search strategy was designed to maximize sensitivity for literature concerning therapeutic and supportive interventions relevant to NVP and HG. Because several antiemetic agents and supportive interventions are also investigated in other nausea and vomiting settings, this broad retrieval strategy could capture a limited number of adjacent-domain records, particularly when pregnancy-related terminology appeared only peripherally or through Web of Science indexing fields. The original corpus was retained for general bibliometric performance analyses, whereas the network-based analyses most susceptible to thematic contamination were recalculated using the refined obstetric subset described below.

Although the condition and intervention blocks were combined with AND, which in principle confines retrieval to a pregnancy-related context, a minority of records centered on adjacent nausea domains were still retrieved. This reflects the deliberate breadth of the query. The condition block included the standalone term “hyperemesis” alongside “hyperemesis gravidarum,” so records on non-obstetric hyperemesis could satisfy it. The Topic field also matches author keywords and Keywords Plus in addition to titles and abstracts, so a record could qualify through index terms that do not appear in its title or abstract. In parallel, the commercial-formulation block retrieves brand-named antiemetic records that do not necessarily restate general pregnancy terminology. Together, these routes account for the limited adjacent-domain periphery observed in the downstream maps.

The final search strategy was structured as follows: ((TS = (“nausea and vomiting of pregnancy” OR “nausea and vomiting in pregnancy” OR “pregnancy nausea” OR “nausea in pregnancy” OR “vomiting in pregnancy” OR “vomiting of pregnancy” OR “emesis in pregnancy” OR “morning sickness” OR “pregnancy sickness” OR “gestational nausea” OR “gestational vomiting” OR “antenatal nausea” OR “antenatal vomiting” OR “prenatal nausea” OR “prenatal vomiting” OR “emesis gravidarum” OR “hyperemesis gravidarum” OR hyperemesis OR “pernicious vomiting” OR disgravidi* OR (NVP NEAR/5 (pregnan* OR gestation* OR gravid* OR hyperemesis OR nausea OR vomit*)) OR (HG NEAR/3 (pregnan* OR gestation* OR gravid* OR hyperemesis)) OR (pregnan* NEAR/10 (nausea OR vomit* OR emesis OR antiemetic*))) AND TS = (pyridoxin* OR “vitamin B6” OR “vitamin-B6” OR doxylamin* OR dimenhydrinat* OR diphenhydramin* OR promethazin* OR meclizin* OR meclozin* OR cyclizin* OR hydroxyzin* OR chlorpheniramin* OR ondansetron* OR metoclopramid* OR domperidon* OR prochlorperazin* OR droperidol* OR “5-HT3 antagonist*” OR “5HT3 antagonist*” OR mirtazapin* OR olanzapin* OR gabapentin* OR antiemetic* OR “anti-emetic*” OR “P6 acupoint” OR “P6 point” OR PC6 OR “PC-6” OR Neiguan OR “Sea-Band*” OR “Sea Band*” OR “enteral nutrition” OR “parenteral nutrition” OR ((ginger OR zingiber OR antihistamin* OR corticosteroid* OR dexamethason* OR methylprednisolon* OR acupunctur* OR acupressure OR cannabi* OR pharmacotherap* OR “intravenous fluid*” OR rehydrat*) NEAR/5 (nausea OR vomit* OR emesis OR hyperemesis OR “morning sickness” OR NVP OR pregnan* OR gestation* OR gravid*)))) OR TS = (“doxylamine-pyridoxine” OR “doxylamine pyridoxine” OR Bendectin OR Debendox OR Diclectin OR Diclegis OR Bonjesta OR Xonvea OR Cariban OR Nacidol OR Navidoxine OR Pregnea OR Emedrin)).

Exact phrase matching was used for compound terms to reduce false-positive retrieval, while wildcard operators were applied to capture morphological variants such as “pyridoxine” and “pyridoxin*”, or “antiemetic” and “antiemetic*”. The search was also designed to avoid ambiguity in the Boolean structure by explicitly enclosing the NVP/HG and intervention blocks in parentheses before adding the commercial formulation block with OR. For clarity, the commercial formulation block was included separately because brand names such as Bendectin, Diclectin, Diclegis, Bonjesta, Xonvea, and Cariban are highly specific to NVP pharmacotherapy. This design reduces the risk of excluding relevant records that mention a formulation name without explicitly repeating general NVP or antiemetic terminology.

To improve the clinical specificity of the network-based analyses, a secondary thematic filter was applied to the initial Web of Science corpus. The original search strategy and the resulting dataset of 1182 unique records were retained for the general bibliometric performance analyses. However, a refined obstetric subset was generated by appending the following exclusion block to the original query: NOT TS = (CINV OR PONV OR (chemotherap* NEAR/5 (nausea OR vomit* OR emesis OR antiemetic*)) OR (postoperativ* NEAR/5 (nausea OR vomit* OR emesis OR antiemetic*)) OR anesthe* OR anaesthe* OR cancer* OR malignan* OR neoplasm* OR oncolog* OR (cannabi* NEAR/3 hyperemesis) OR (marijuana NEAR/3 hyperemesis) OR “cyclic vomiting syndrome”). Because the Topic field includes titles, abstracts, Author Keywords, and Keywords Plus, this secondary filter removed records centered on chemotherapy-induced nausea and vomiting, postoperative nausea and vomiting, oncology, anesthesia, cannabinoid or cannabis hyperemesis, and cyclic vomiting syndrome. The refined search yielded 709 records, which were used exclusively to calculate the country collaboration network, keyword co-occurrence network, and thematic evolution analysis. All other bibliometric indicators remained based on the original 1182-record corpus.

**Figure 1:**
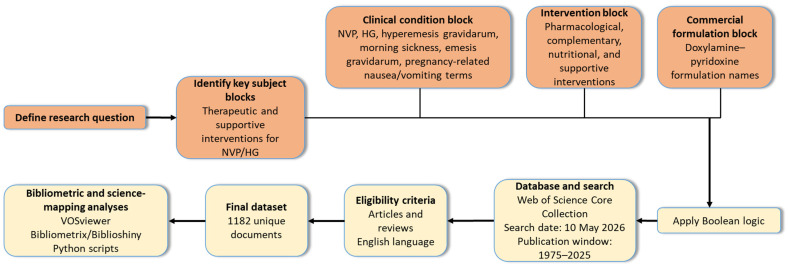
Workflow of the Web of Science search, filtering, and bibliometric analysis process. The search strategy was structured around clinical condition, intervention, and commercial formulation concept blocks. Retrieved records were filtered by document type and language, deduplicated to obtain the final dataset, and analyzed using VOSviewer v1.6.20, Bibliometrix R 4.2, and Python v3.12.3 scripts.

The initial WoSCC retrieval was refined using predefined eligibility criteria. Only documents classified as articles or review articles were retained. Editorials, letters, meeting abstracts, proceedings papers, book chapters, corrections, news items, and other non-research document types were excluded. The language was restricted to English to ensure consistency in keyword extraction, terminology standardization, and thematic interpretation. Retrieved records were exported from WoSCC, merged into a single dataset, and deduplicated through a multi-stage verification process. Records were first matched by their Web of Science accession number (UT), followed by their DOI where necessary. For any remaining ambiguous entries, a combined criterion of normalized title and publication year served as the final resolution mechanism. The final dataset comprised 1182 unique documents. The complete search, filtering, deduplication, and analysis workflow is summarized in Figure 1.

To provide an empirical basis for evaluating the coverage implications of the single-database design, the Web of Science search strategy was additionally adapted for execution in PubMed and Scopus. Because these platforms differ from the Web of Science Core Collection in field structure, phrase indexing, and proximity operator syntax, the original query could not be reproduced identically and was instead translated into database-specific equivalents. In Scopus, the topic terms were mapped to the TITLE-ABS-KEY field with the proximity operators adjusted to the corresponding Scopus syntax. In PubMed, the terms were restricted to the title and abstract fields, with language and document-type limits applied through the corresponding filters. The same eligibility criteria used for the primary corpus were retained across all three databases, namely English-language articles and review articles published from 1975 to 2025. This procedure was performed solely to obtain approximate, order-of-magnitude record counts for cross-database comparison and did not involve record-level merging or deduplication. The complete search strings used across all three databases are compiled in the Supplementary Materials (Table S1) to support reproducibility.

### 2.2. Data Export, Merging, Deduplication, and Software Environment

After application of the search strategy and eligibility filters, the retrieved records were exported from the Web of Science Core Collection in tab-delimited text format using the “Full Record and Cited References” export option. This format was selected to preserve the bibliographic, citation, affiliation, abstract, keyword, and cited-reference metadata required for performance analysis, collaboration mapping, keyword analysis, and thematic evolution. When Web of Science divided the export into multiple files, all files were imported and merged into a single working dataset while preserving the original field structure.

The merged dataset was screened for technical inconsistencies before analysis. Repeated header rows, empty identifier fields, and invalid publication-year values were checked, and publication years were extracted from the PY field and converted to numeric format. To resolve duplicate records, we implemented a multi-tiered matching protocol: the Web of Science accession number (UT) served as the main identifier, supplemented by the DOI, while a combination of normalized titles and publication years was applied as a final verification step. Title normalization included lowercasing, punctuation removal, whitespace standardization, and correction of formatting inconsistencies. Although this deduplication workflow was applied systematically, no duplicate records were identified in the final export; therefore, the dataset retained 1182 unique documents for analysis.

Bibliometric processing and science-mapping analyses were performed using VOSviewer version 1.6.20, the Bibliometrix R 4.2 package and its web interface Biblioshiny, together with custom Python (3.12.3) scripts for data cleaning, changepoint detection, metadata-completeness assessment, and keyword normalization. VOSviewer was used for network visualizations, including country collaboration and keyword co-occurrence maps, while Bibliometrix/Biblioshiny was used for complementary bibliometric indicators and thematic evolution analysis. Custom Python scripts were used to ensure reproducible preprocessing, deduplication checks, temporal analyses, and thesaurus generation [15,16,17].

### 2.3. Bibliometric Indicators and Performance Analysis

Country names were extracted from the dataset using VOSViewer and systematically reviewed to identify unique country labels and spelling variants. After inspection, only clear country-name variants were normalized, while Web of Science/VOSviewer territorial labels such as England, Scotland, and Wales were retained separately to preserve consistency with the source database output. The thesaurus therefore harmonized alternative or database-specific labels by mapping “Peoples R China” and “People’s R China” to “China,” and “Turkiye” to “Turkey”. No broader geopolitical aggregation was applied beyond these explicit name harmonizations.

A metadata-completeness sensitivity analysis was performed to assess whether historical changes in Web of Science indexing could influence downstream topic-based analyses. This step was included because the Web of Science Topic field depends not only on titles, but also on abstracts, author keywords, and Keywords Plus, whose availability may vary across older records. The analysis was implemented using a custom Python script applied to the merged Web of Science export file. The script detected the main bibliographic fields used in the analysis, including publication year (PY), title (TI), abstract (AB), author keywords (DE), Keywords Plus (ID), DOI (DI), and Web of Science accession number (UT).

Binary completeness indicators were generated for title, abstract, author keywords, and Keywords Plus. The analysis calculated yearly metadata coverage and period-level coverage before and after 1991, with the comparison defined as pre-1991 versus 1991-and-later records. Statistical comparisons of metadata availability between the two periods were performed using two-sided Fisher’s exact tests.

For graphical evaluation, years with fewer than five records were treated as unstable annual estimates and were shown as low-n points rather than interpreted as reliable yearly trends. The script generated yearly metadata-completeness plots, pre/post-1991 period-comparison plots with Wilson confidence intervals, summary tables, statistical checks, parsing logs, and a cleaned metadata dataset. The results of this sensitivity analysis were used to determine whether keyword-based analyses could be interpreted across the full study period or should be restricted to records published from 1991 onward.

The organization counts were based on the Web of Science C3 organization-enhanced field and used full counting, with each organization counted once per document-year.

### 2.4. PELT Changepoint Detection and Parameter Sensitivity Analysis

To identify statistically supported transitions in annual publication activity, changepoint detection was performed on the yearly document-count series. Web of Science tab-delimited export files were imported into Python, and records were deduplicated using the Web of Science unique identifier field (UT). Publication years were extracted from the PY field, and annual production was calculated as the number of unique records per year. The final time series comprised 51 consecutive annual observations covering 1975–2025 and included 1182 unique documents. All calendar years within this interval were represented in the dataset.

Changepoints were detected using the Pruned Exact Linear Time (PELT) algorithm implemented in the Python ruptures package. The annual document-count series was analyzed as a univariate signal using the radial basis function (rbf) cost model. The primary macrotrend analysis used a minimum segment size of three years and a candidate-position step of jump = 5, thereby evaluating potential breakpoint locations at five-observation intervals. This specification was used to identify broad temporal segmentation across the five-decade publication series, while annual-resolution localization was examined separately in the parameter-sensitivity analysis described below.

The RBF cost was selected because it is a non-parametric kernel-based model capable of detecting broader changes in the distribution of the annual publication series rather than being restricted to changes in a single predefined parameter. A standard least-squares mean-change model would primarily identify shifts between piecewise-constant mean levels, whereas a linear cost model would require an explicitly specified regression structure and would focus on changes in regression coefficients. Because the five-decade publication series showed sustained growth, irregular annual fluctuations, and potentially changing dispersion, no assumption was made that relevant transitions would consist exclusively of abrupt mean shifts or piecewise-linear slope changes. The RBF specification was therefore used to identify broad changes in publication regimes, without attributing clinical causality to the resulting changepoints.

A minimum segment size of three years was predefined as the shortest interval considered sufficient to represent a sustained bibliometric phase rather than an isolated annual fluctuation. This parameter does not average, aggregate, or smooth the annual publication counts. Instead, it requires at least three observations between adjacent changepoints. Consequently, a persistent change in publication activity may still be localized to its year of onset, whereas a fluctuation lasting only one or two years cannot form an independent segment. The three-year minimum was selected to reduce over-segmentation and false-positive changepoints arising from random annual variability, particularly during the early decades of the series when publication output was sparse and irregular. This specification therefore prioritized the identification of sustained five-decade publication patterns over transient responses confined to one or two publication years.

Changepoint stability was initially evaluated across ten penalty values ranging from 1.0 to 10.0. For each penalty setting, the detected breakpoints were converted into calendar years and reported as the first year of the subsequent segment. An exact-year changepoint was considered robust when it was detected under at least 50% of the evaluated penalty settings, corresponding to a minimum of five out of ten runs.

To determine whether the predefined minimum segment size reduced sensitivity to rapid changes, an annual-resolution sensitivity analysis was conducted in which every year was evaluated as a potential changepoint (jump = 1). Minimum segment sizes of one, two, and three years were tested across penalty values of 1.0–10.0 while retaining the radial basis function (rbf) cost model, resulting in 30 parameter combinations. This analysis was used as a quality-control procedure rather than as a replacement for the primary macrotrend segmentation conducted with jump = 5. It assessed whether the broader temporal transition pattern remained evident when breakpoint localization was permitted at individual years. Breakpoint locations and detection frequencies were compared across the three minimum-segment specifications, and nearby detected years were interpreted descriptively as broader transition windows. Complete results from all parameter combinations are provided in Supplementary Table S2.

For descriptive period comparisons and thematic-evolution analysis, the broader temporal transition pattern observed across the PELT penalty grid was operationalized into three analytical macroperiods: 1975–1994, 1995–2009, and 2010–2025. The cut points were selected within the mid-1990s and late-2000s/early-2010s transition windows observed across the PELT penalty grid. These macroperiod boundaries were not interpreted as exact annual changepoint estimates. Rather, they provided intervals of broadly comparable duration with sufficient document and keyword density for comparing long-term publication patterns and keyword structures without over-interpreting small differences in exact breakpoint localization.

For each macroperiod, descriptive statistics were calculated, including the number of years, total documents, mean annual production, median annual production, and standard deviation. Adjacent macroperiods were compared using the Mann–Whitney U test because annual publication counts were not assumed to be normally distributed. Effect sizes were estimated using Hedges’ g and rank-biserial correlation. The PELT trend plot, growth-rate plot, macroperiod boxplot, sensitivity tables, detection-frequency outputs, and period-comparison results were generated using Python libraries including pandas 2.3.2, numpy 2.3.3, scipy 1.16.2, ruptures 1.1.10, matplotlib 3.10.6, and seaborn 0.13.2 [18,19,20,21,22].

### 2.5. Keyword Normalization and Thesaurus Validation

**Figure 2:**
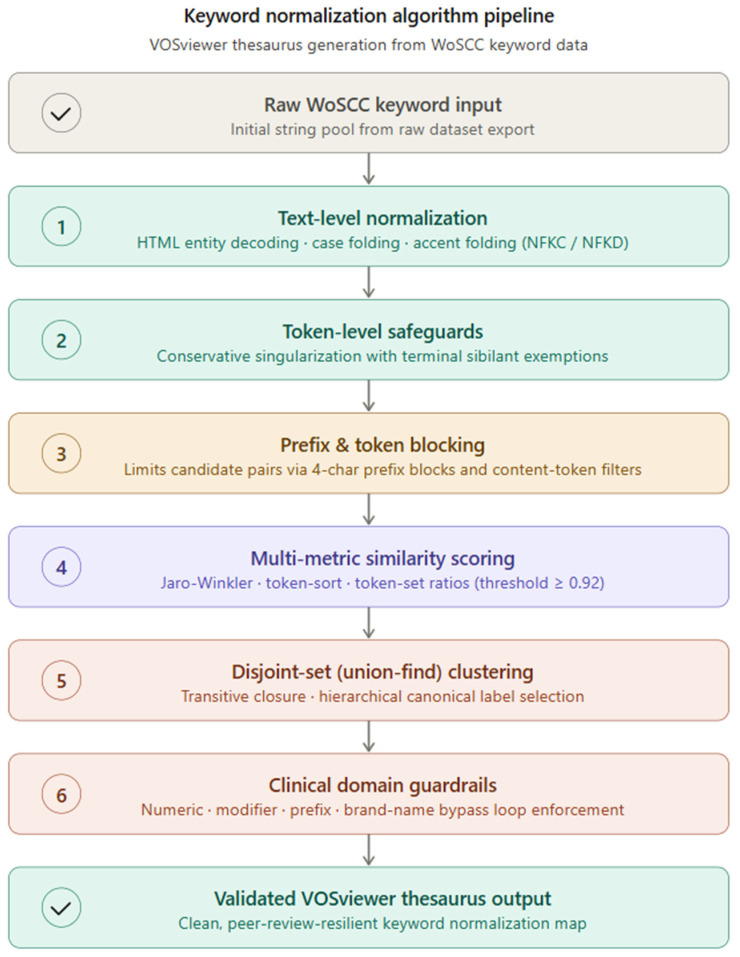
Algorithmic schema of the multi-metric string-similarity and domain-guardrail framework.

Author-supplied keywords and Keywords Plus terms were consolidated into an optimized science-mapping thesaurus using a custom preprocessing pipeline executed in Python 3.12.3. Text strings were subjected to uniform normalization, including HTML entity decoding, case folding, accent stripping via Unicode canonical decomposition (NFKC/NFKD), and targeted token-level singularization backed by explicit clinical exemptions for terminal sibilants (e.g., nausea, hyperemesis, cannabis). To eliminate computationally intensive all-pairs comparisons, candidate linkages were restricted using a four-character prefix and token-co-occurrence blocking architecture. Surviving pairs were evaluated across three complementary similarity dimensions: character-level Jaro–Winkler similarity via the jellyfish (v1.2.1) library [23], alongside token-sort and token-set ratios computed via the standard library’s difflib.SequenceMatcher. Pairs demonstrating a maximum similarity score of 0.92 or higher, exact normalized variants, recognized domain abbreviation expansions (e.g., NVP, HG), and curated clinical synonym blocks were linked deterministically. Linked terms were clustered via transitive closure using a disjoint-set (union–find) structure, with canonical preferred labels automatically selected based on historical curation status, formatting cleanliness, and raw dataset frequency. The complete systematic mechanism of this computational pipeline is illustrated in Figure 2.

To preserve downstream thematic precision, strict domain guardrails were enforced at generation time and re-verified on every final emitted path to block semantically unsafe transitive merges. Pairings were automatically rejected if they introduced numeric or Roman numeral mismatches (e.g., phase II vs. phase III), protected clinical modifier conflicts (mild vs. severe), contrastive semantic token changes (abuse vs. use), or flipped meanings via negating prefixes (malnutrition vs. nutrition). Single-token substitutions between distinct nouns or clinical codes (e.g., maternal age vs. maternal use; QTc vs. QT) and the dropping of critical diagnostic context markers were similarly blocked. To completely protect the structural integrity of the main corpus network, the pipeline skipped the automatic merging of commercial drug trade names (e.g., Bendectin, Diclectin, Diclegis). Instead of allowing these proprietary formulations to map directly onto generic compounds (doxylamine pyridoxine) via automated clustering, they were systematically isolated and diverted to a separate manual-review register to eliminate the risk of automatic cluster pollution, while invalid mappings were formally pruned.

Rather than sampling, a full census of all 253 unique generated candidate mappings was subjected to a blind, multi-investigator consensus review across separate baseline, high-stringency, and brand-to-generic evaluation vectors. Mappings were classified as good if they accurately preserved the underlying biomedical concept or bad if they collapsed distinct clinical entities, with discrepancies reconciled via a majority-vote rule. The pipeline achieved an empirical macro-level acceptance rate of 97.63% (247 out of 253; 95% Wald confidence interval: 95.75% to 99.51%), significantly exceeding the self-imposed 90% quality threshold. Investigators demonstrated a mean pairwise raw agreement of 85.50%. Due to the high prevalence of the good classification (approximately 98%), chance-corrected indicators like Cohen’s kappa [24] and Fleiss’ kappa [25] were heavily suppressed by the well-documented prevalence paradox [26]. Consequently, Gwet’s AC1 coefficient was utilized as the primary stability indicator [27], yielding a value of 0.832. This indicates almost perfect inter-rater reliability and confirms the structural integrity of the final validated VOSviewer thesaurus.

### 2.6. Network and Science-Mapping Analyses

The country collaboration network, keyword co-occurrence network, and thematic evolution analysis were conducted using the refined obstetric subset generated through the secondary thematic filtering procedure described in Section 2.1. The country collaboration network map represents nodes as countries, while the size of the node is directly proportional to the number of the documents published, the color indicates the cluster, and the width of the line connecting two countries is directly proportional with the collaboration strength of those countries. Only countries that have at least one active collaboration are depicted.

To map the foundational thematic architecture of the literature, we conducted a global keyword co-occurrence analysis across the entire timeframe of the dataset using VOSviewer. The network structure integrated both Keywords Plus and Author Keywords extracted from the Web of Science records. We restricted the final visualization to high-frequency terms by enforcing a strict minimum threshold of ten occurrences. Within the generated network diagram, thematic clusters are distinguished by color, the frequency of a keyword dictates its node size, and the proximity/thickness of the connections reveals the relative strength of co-occurrence between concepts. Because the metadata-completeness sensitivity analysis showed limited availability of Author Keywords and Keywords Plus before 1991, the resulting all-years keyword map was interpreted as an exploratory corpus-level structure rather than as a temporally balanced thematic representation.

Thematic evolution was examined using Bibliometrix/Biblioshiny based on all available Web of Science keyword fields, including Author Keywords and Keywords Plus. Keywords were analyzed across three broad analytical macroperiods: 1975–1994, 1995–2009, and 2010–2025. These intervals were informed by the mid-1990s and late-2000s/early-2010s transition windows observed in the PELT parameter-sensitivity analysis but were not treated as exact changepoint estimates. The broad intervals were retained to ensure sufficient document and keyword density within each period and to support interpretable comparison across the five-decade study horizon. Thematic links between periods were generated using shared keywords, and the resulting Sankey-type map was used to visualize thematic continuity, transition, and diversification. Because the metadata-completeness sensitivity analysis showed limited Author Keyword and Keywords Plus coverage before 1991, the first macroperiod was interpreted cautiously.

Author co-citation analysis was performed using the refined 709-record obstetric subset and the cited-reference metadata exported from WoSCC. In VOSviewer, co-citation was selected as the analysis type, cited authors as the unit of analysis, and full counting as the counting method. A minimum threshold of 40 citations per cited author was applied to retain the most frequently cited contributors while preserving an interpretable network structure. Forty-eight cited authors met this threshold, and all 48 formed a single interconnected network. In the resulting visualization, node size represents the number of citations received by each cited author, link width reflects the strength of co-citation relationships, and node color indicates the cluster assigned by VOSviewer.

## 3. Results

### 3.1. Growth Analysis of Global Publication Output and Citation Dynamics

**Figure 3:**
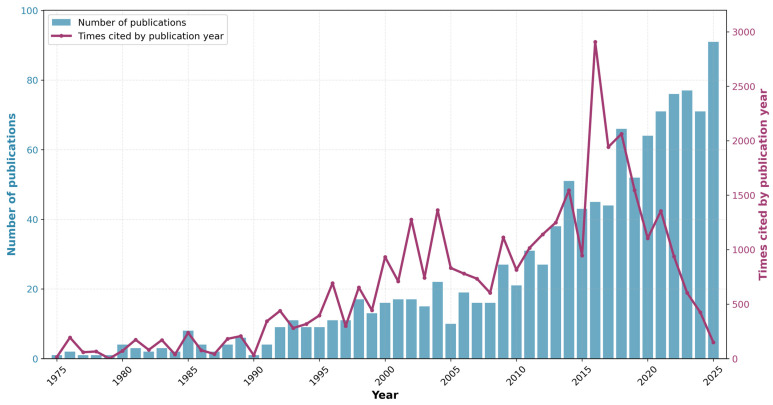
Temporal evolution of publication output and citation accumulation in research on therapeutic and supportive interventions for nausea and vomiting of pregnancy and hyperemesis gravidarum from 1975 to 2025. Bars indicate the annual number of publications, while the line represents the cumulative Web of Science times-cited count for documents published in each year. Citation values reflect current cumulative citations by publication year and should not be interpreted as citations received within that calendar year.

The final dataset comprised 1182 unique publications indexed between 1975 and 2025, showing a clear long-term expansion of research on therapeutic and supportive interventions for nausea and vomiting of pregnancy and HG (Figure 3). Annual output was initially low and irregular, with only 45 documents published between 1975 and 1990, followed by gradual growth during 1991–2000 (110 documents) and further consolidation during 2001–2010 (180 documents). The most pronounced expansion occurred after 2011, with 847 publications produced between 2011 and 2025, representing 71.7% of the entire dataset. Publication activity remained consistently elevated from 2018 onward and reached its maximum in 2025, with 91 documents. Citation dynamics followed a different pattern, as the values represent cumulative citations received by documents published in each year rather than citations accumulated during that calendar year. The highest citation peak was observed for the 2016 publication cohort, with 2908 citations, followed by 2018 with 2063 citations and 2017 with 1942 citations. Although recent years, particularly 2022–2025, show lower citation counts, this mainly reflects the shorter citation window available for newer publications rather than reduced scientific relevance. Overall, the temporal profile indicates a transition from sporadic early publication activity to sustained and accelerating scientific production, while citation patterns suggest that several cohorts from the 2000s and mid-2010s have had particularly strong influence in the field.

The 2016 cohort represented the most notable recent citation peak, combining substantial publication volume with the strongest post-2010 mean citation impact. After 2021, MeanTCperArt declined progressively, from 19.08 in 2021 to 12.33 in 2022, 7.83 in 2023, 5.99 in 2024, and 1.63 in 2025. This downward trend should be interpreted mainly as a citation-window effect, since recent publications have had fewer citable years, declining from 50 years for the 1975 cohort to 0 years for 2025, rather than as evidence of reduced scientific relevance.

**Figure 4:**
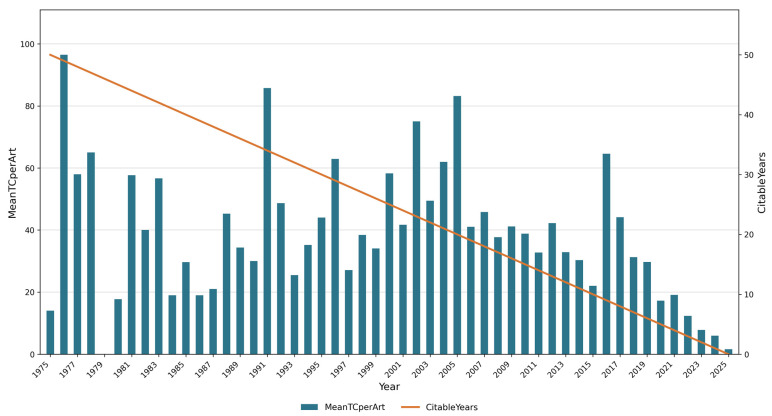
Mean citation impact and citable years for publications on therapeutic and supportive interventions for nausea and vomiting of pregnancy and hyperemesis gravidarum from 1975 to 2025. Bars represent the mean number of Web of Science times cited per article for each publication year, while the line indicates the number of citable years calculated relative to 2025. Recent publication cohorts should be interpreted cautiously because they have had less time to accumulate citations.

Mean citation impact by publication year showed substantial variability across the study period, reflecting both differences in scientific influence and the unequal time available for citation accumulation (Figure 4). The highest MeanTCperArt was observed in 1976, with 96.50 citations per article, although this value should be interpreted cautiously because it was based on only two publications. Other high-impact cohorts were identified in 1991 (85.75), 2005 (83.20), 2002 (75.06), 2016 (64.62), 1996 (62.91), and 2004 (61.95). Compared with the earliest decades, where annual values were strongly affected by small publication counts, the 1995–2010 interval showed a more stable citation profile, with several years exceeding 40 citations per article and multiple cohorts surpassing 60 citations per article.

### 3.2. Geographic Distribution of Research Output

The geographic distribution of publications showed a clear predominance of the United States, which contributed 519 documents and accumulated 18,036 citations, representing the highest output and citation burden among all countries included in the analysis (Table 1). Canada ranked second by publication volume, with 144 documents and 4298 citations, followed by England with 84 documents and 3899 citations, and Australia with 66 documents and 4188 citations.

When citation efficiency was considered, the hierarchy differed from the publication-volume ranking. Italy recorded the highest average citation rate, with 74.46 citations per document, despite contributing only 37 publications. Australia also showed a strong citation profile, with 63.45 citations per document, followed by Germany (60.33), China (50.59), and England (46.42). In contrast, Brazil and India showed lower average citation rates, with 7.59 and 21.93 citations per document. Overall, the results indicate that the field is led quantitatively by the United States, while several countries with lower publication volumes, particularly Italy, Australia, Germany, China, and England, demonstrate substantial citation impact relative to output.

Although an English-language criterion was specified in the eligibility rules, it had no filtering effect on the retrieved corpus: all 1182 records returned by the search strategy were already published in English, so the language restriction excluded no documents. The country-level distribution reported above therefore reflects the retrieved Web of Science literature directly, without an additional language-based exclusion step, and the English criterion cannot be a source of differential geographic bias within the analyzed dataset. Any residual under-representation of non-English research would instead arise at the level of database indexing rather than the language criterion.

**Figure 5:**
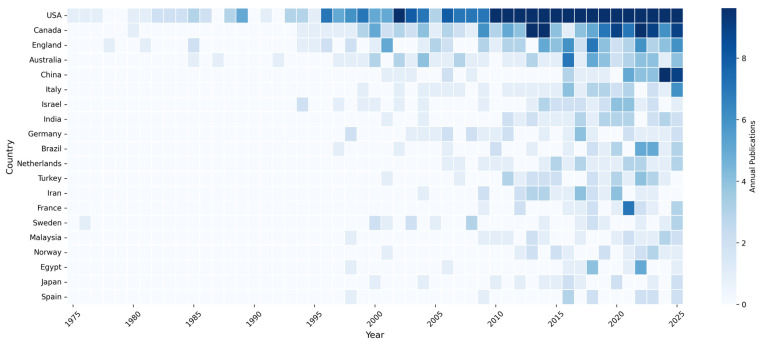
Longitudinal heatmap of country-attributed publication activity in NVP/HG therapeutic and supportive intervention research from 1975 to 2025. Each cell represents the number of publications attributed to a given country in a specific year, based on Web of Science author-address data and full counting. Darker blue shading indicates higher annual output. Countries are listed vertically, while publication years are shown horizontally at five-year intervals. The visualization was generated in Python using the Seaborn and Matplotlib libraries.

The temporal heatmap adds a different perspective by showing how national participation evolved across the study period (Figure 5). In the early decades, country-attributed output was sparse and concentrated mainly in the USA, with isolated contributions from England, Canada, Australia, and Sweden. A broader international pattern began to emerge after the late 1990s, when additional countries started appearing more consistently in the annual output. The diversification became more visible after 2010, with repeated contributions from European, Middle Eastern, Asian, and Latin American countries. In the most recent period, the heatmap shows particularly strong activity from the USA, sustained participation from Canada and England, and a late but visible acceleration in China, especially after 2021. Thus, while Table 1 identifies the leading countries by cumulative output and impact, Figure 5 shows the chronological transition from a concentrated research landscape toward a more internationally distributed field.

### 3.3. Publication Venues and Institutional Contributions

The source-impact analysis showed that the literature on therapeutic and supportive interventions for nausea and vomiting of pregnancy and HG is concentrated mainly in obstetrics, gynecology, reproductive safety, and pregnancy-related clinical journals (Table 2). Obstetrics and Gynecology was the leading source, with the highest h-index (24), g-index (39), total citations (1971), and number of publications (39). American Journal of Obstetrics and Gynecology ranked second, with a comparable impact profile, including an h-index of 23, a g-index of 28, 1396 citations, and 28 publications. These two journals represent the main publication venues in the field and reflect the strong anchoring of NVP/HG intervention research within obstetric and maternal medicine.

Beyond these two dominant journals, the publication landscape was more diversified. Reproductive Toxicology and Teratology highlight the importance of fetal safety and drug-exposure assessment, while BMC Pregnancy and Childbirth, Journal of Maternal-Fetal & Neonatal Medicine, and American Journal of Perinatology reflect the broader maternal–fetal and perinatal orientation of the field. The m-index, or m-quotient, is defined as a source’s h-index divided by the number of years since its first publication in the analyzed corpus, normalizing citation impact for the length of time a source has contributed to the field and allowing more recent sources to be compared with long-established ones [28]. BMC Pregnancy and Childbirth had the highest m-index (0.643), despite beginning later in the dataset, suggesting a relatively rapid accumulation of source-level influence. The presence of American Family Physician, American Journal of Emergency Medicine, and Neurogastroenterology and Motility further indicates that the topic extends beyond specialist obstetrics into primary care, emergency medicine, and gastrointestinal research. Overall, publication venues suggest a clinically oriented and multidisciplinary field, with core leadership from obstetrics journals and secondary contributions from safety, perinatal, primary care, emergency, and gastrointestinal sources.

**Figure 6:**
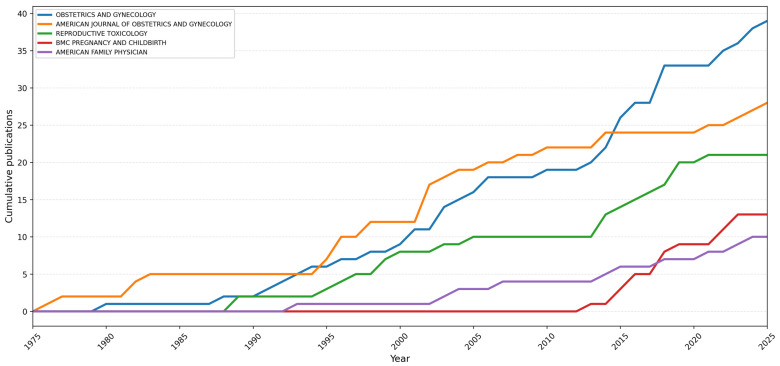
Cumulative publication trajectories of the five leading journals in NVP/HG therapeutic and supportive intervention research from 1975 to 2025.

The cumulative publication trajectories of the five leading journals show that the dissemination of NVP/HG therapeutic and supportive intervention research evolved through distinct source patterns (Figure 6). American Journal of Obstetrics and Gynecology contributed early and steadily, with visible accumulation from the late 1970s and a strong increase around 2002, but its curve became relatively stable after the mid-2010s. Obstetrics and Gynecology showed slower initial growth, then accelerated markedly after 2014, overtaking the other sources and reaching the highest cumulative output by 2025. Reproductive Toxicology followed a more gradual trajectory beginning in 1989, consistent with its narrower focus on drug exposure and reproductive safety. In contrast, BMC Pregnancy and Childbirth entered the dataset only after 2013 but accumulated publications rapidly during the following decade, reflecting the growing role of open-access pregnancy-focused journals. American Family Physician showed a lower but persistent contribution, indicating that NVP/HG management also remains relevant to primary care practice. Overall, the figure suggests a shift from early concentration in traditional obstetric journals toward a broader publication landscape involving safety-focused, open-access, and frontline clinical sources.

**Figure 7:**
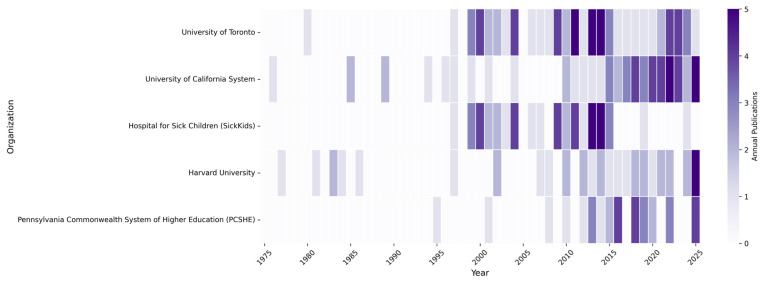
Annual publication activity of the leading organizations in NVP/HG therapeutic and supportive intervention research from 1975 to 2025. The heatmap displays annual publication counts for the five most productive organizations, based on the Web of Science organization-enhanced field. Darker purple shading indicates higher annual output. Organization counts use full counting, with each organization counted once per document-year, regardless of repeated appearances within the same record.

The institutional heatmap should be interpreted as a temporal activity pattern rather than a static institutional ranking. The annual organization-level distribution shows that the University of Toronto and Hospital for Sick Children (SickKids) formed the clearest early-to-mid period institutional axis, with recurrent activity from the late 1990s and a shared peak in 2013, when both reached 8 publications (Figure 7). This pattern is consistent with a strong Canadian contribution to the field and suggests that NVP/HG intervention research was partly shaped by a concentrated maternal–child health research environment.

In contrast, the University of California System showed a more recent and progressively intensifying pattern, especially after 2015, reaching its highest annual output in 2025 with 7 publications. Harvard University contributed intermittently across the full study period, with early activity in the late 1970s and 1980s and a renewed peak in 2025 with 5 publications. The Pennsylvania Commonwealth System of Higher Education appeared later and showed episodic activity, particularly in 2016, 2018, and 2025. Overall, the heatmap indicates a shift from concentrated Canadian institutional leadership toward broader North American institutional participation in the most recent decade.

### 3.4. International Collaboration and Conceptual Structure

**Figure 8:**
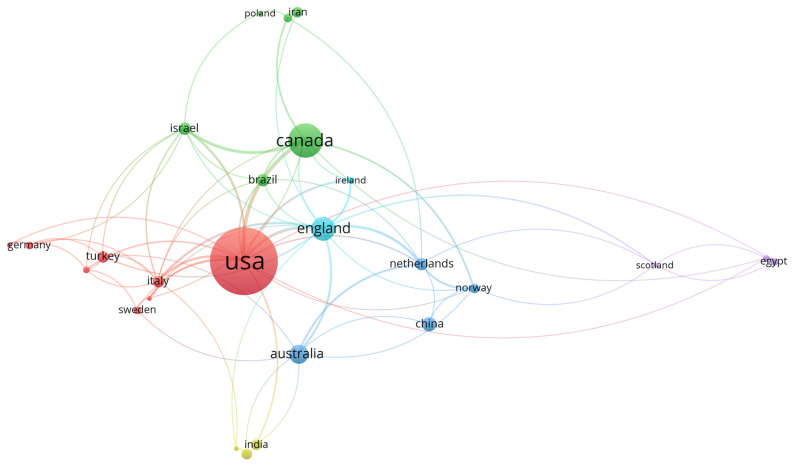
International country collaboration network in NVP/HG therapeutic and supportive intervention research. The visualization scales country nodes relative to total publication output and determines line thickness based on the intensity of cross-border partnerships. Thematic groupings of collaborating nations are differentiated by distinct color clusters.

The country collaboration network based on the refined obstetric subset identified the USA as the principal international collaboration hub (Figure 8). The USA contributed 244 documents and showed both the highest number of direct country links (15) and the highest total link strength (TLS = 52). England occupied the second most central position, with 14 links and a TLS of 40, followed by Canada, with 10 links and a TLS of 34. The strongest bilateral collaboration was observed between Canada and the USA (link strength = 13), followed by England–USA (9), Canada–Israel (8), Egypt–Saudi Arabia (7), and England–Netherlands (6). Other notable partnerships included England–Ireland, England–Italy, Israel–USA, and Italy–USA, each with a link strength of 5.

The network was organized into six collaboration clusters. One cluster included the USA together with Italy, Germany, Denmark, Sweden, Spain, Turkey, and South Korea. A second cluster was structured around Canada and Israel and also included Brazil, France, Iran, and Poland. Australia, the Netherlands, Norway, and China formed another collaboration group, while smaller regional clusters linked Egypt, Saudi Arabia, and Scotland; India, Indonesia, and Malaysia; and England and Ireland. Although England and Canada belonged to separate clusters, both maintained substantial connections across the wider network. Overall, the refined analysis shows that international collaboration in NVP/HG therapeutic and supportive-intervention research remains anchored primarily around the USA, England, and Canada, with additional regional partnerships contributing to a broader but comparatively less intensive global network.

### 3.5. Changepoint-Based Temporal Segmentation

Under the primary macrotrend specification (jump = 5, min_size = 3), PELT identified two publication-phase boundaries at 1995 and 2010. The 2010 breakpoint was detected under all ten penalty settings, while the 1995 breakpoint was detected under five of ten settings, satisfying the predefined robustness criterion. These boundaries defined three broad macroperiods: 1975–1994, 1995–2009, and 2010–2025.

The annual-resolution sensitivity analysis (jump = 1) showed that exact breakpoint localization varied within broader mid-1990s and early-2010s transition windows. The year 2013 was detected under six of ten penalty settings for each tested minimum segment size, while 2011 was detected under the remaining four higher-penalty settings. Minimum segment sizes of two and three years produced identical results across all penalties, and allowing one-year segments did not reveal a breakpoint in 1983. These findings indicate that min_size = 3 did not obscure the principal recent transition, although the precise annual location depended on candidate-position resolution.

**Figure 9:**
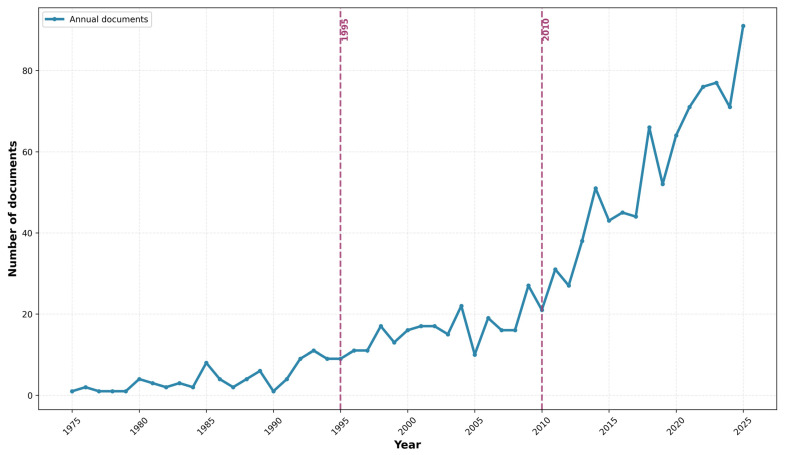
PELT-based changepoint detection of annual publication output in NVP/HG therapeutic and supportive intervention research from 1975 to 2025. The line represents annual publication counts, while dashed vertical lines indicate robust changepoints detected by PELT sensitivity analysis. The identified breakpoints define three publication phases: 1975–1994, 1995–2009, and 2010–2025. These boundaries define three broad analytical macroperiods: 1975–1994, 1995–2009, and 2010–2025. Annual-resolution localization and minimum-segment sensitivity results are reported in Supplementary Table S2.

PELT changepoint analysis identified two robust publication-phase boundaries, corresponding to 1995 and 2010 (Figure 9). The 2010 breakpoint was the most stable transition, being detected across all ten penalty settings, whereas the 1995 breakpoint was detected in five of ten settings and therefore represents a threshold-level robust change. These breakpoints divided the 1975–2025 publication series into three periods: an early low-output phase from 1975 to 1994, an intermediate consolidation phase from 1995 to 2009, and a high-output expansion phase from 2010 to 2025. Mean annual production increased from 3.90 documents in Period 1 to 15.73 in Period 2 and 54.25 in Period 3. Adjacent-period comparisons confirmed statistically distinct publication levels, with very large effect sizes for both transitions: Period 1 vs. Period 2, *p* = 1.119 × 10^−6^, Hedges’ g = 2.986; Period 2 vs. Period 3, *p* = 3.657 × 10^−6^, Hedges’ g = 2.518. These findings support a data-driven temporal segmentation of the field, indicating a shift from sporadic early output to sustained growth after 1995 and marked acceleration after 2010.

### 3.6. Metadata Completeness and Indexing Sensitivity

**Figure 10:**
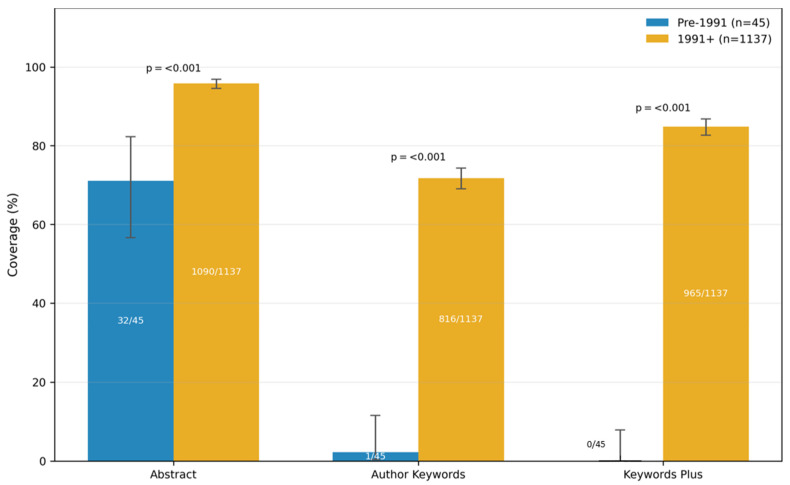
Metadata-completeness sensitivity analysis before and after 1991. Bars show the proportion of records containing abstracts, author keywords, and Keywords Plus in the pre-1991 and 1991-and-later subsets of the NVP/HG therapeutic and supportive intervention corpus. Error bars represent 95% Wilson confidence intervals, and *p*-values were calculated using two-sided Fisher’s exact tests. The marked post-1991 increase in author keyword and Keywords Plus coverage demonstrates that keyword-based and thematic analyses spanning the pre-1991 period require cautious, exploratory interpretation rather than direct comparison with later periods.

Marked differences in metadata availability were observed between the pre-1991 and 1991-and-later records. Among records published before 1991, abstract coverage was 71.1%, author keyword coverage was 2.2%, and Keywords Plus coverage was 0.0%. In contrast, among records published from 1991 onward, abstract coverage increased to 95.9%, author keyword coverage to 71.8%, and Keywords Plus coverage to 84.9%. The magnitude and uncertainty of these pre/post-1991 differences are summarized in Figure 10.

Fisher’s exact tests confirmed that these differences were statistically significant for all evaluated metadata fields. Abstract availability increased from 32/45 records before 1991 to 1090/1137 records from 1991 onward, corresponding to a difference of +24.76 percentage points (*p* < 0.001). Author keyword availability increased from 1/45 to 816/1137 records, a difference of +69.55 percentage points (*p* < 0.001). Keywords Plus availability increased from 0/45 to 965/1137 records, a difference of +84.87 percentage points (*p* < 0.001). The proportion of records containing all three non-title Topic-field components increased from 0.0% before 1991 to 61.9% from 1991 onward, while the proportion missing all three components decreased from 28.9% to 0.9%.

These findings indicate that Topic-field completeness changed substantially around the early 1990s. Title coverage was complete across the full period, confirming that title-based analyses are entirely unaffected by this indexing discontinuity. Abstract-based analyses can similarly be interpreted across the full 50-year range, provided the reduced pre-1991 baseline coverage is acknowledged. In contrast, because author keywords and Keywords Plus are nearly absent before 1991, subsequent long-range keyword co-occurrence, thematic evolution, and trend-topic analyses spanning the entire study period must be interpreted with strict caution. Rather than reflecting immediate thematic diversification or sudden historical shifts in research focus, the rapid expansion of keyword nodes after 1991 is partially an artifact of this database indexing change. Consequently, while all-years visualizations are retained to capture global macro-level conceptual structures, their early chronological segments are treated as exploratory bibliometric signals rather than a balanced historical record.

### 3.7. Keyword Co-Occurrence and Thematic Evolution

**Figure 11:**
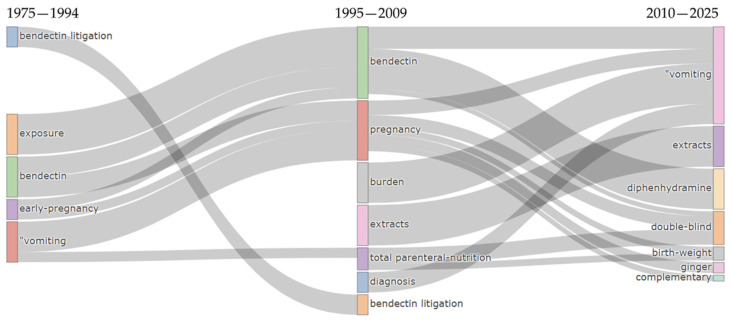
Thematic evolution map of the refined obstetric subset across the three publication periods identified by PELT analysis of the complete corpus: 1975–1994, 1995–2009, and 2010–2025. The Sankey-type map was generated using the available Web of Science Author Keywords and Keywords Plus. Nodes represent the dominant themes within each period, while the width of the connecting flows indicates thematic continuity based on shared keywords between consecutive periods. The first period should be interpreted cautiously because of the limited availability of keyword metadata before 1991.

The thematic evolution analysis of the refined obstetric subset showed a clear progression from early concerns regarding pregnancy-related vomiting and medication safety toward a broader clinical and therapeutic research structure (Figure 11). During 1975–1994, the thematic landscape was dominated by vomiting, Bendectin, Bendectin litigation, early pregnancy, and exposure. These themes reflect the historical importance of fetal-safety concerns, teratogenic-risk assessment, and the scientific and legal controversy surrounding Bendectin. The strongest transition from the first to the second period was observed between early pregnancy and pregnancy, with five shared keyword occurrences, followed by vomiting to pregnancy and early pregnancy to Bendectin, each with four occurrences. Bendectin, exposure, and Bendectin litigation also showed continuity into the 1995–2009 period.

During 1995–2009, pregnancy and Bendectin became the principal organizing themes, accompanied by burden, diagnosis, total parenteral nutrition, and continuing Bendectin litigation. In the final period, 2010–2025, the pregnancy theme diversified most strongly toward vomiting, with 36 shared keyword occurrences, double-blind research with 15 occurrences, and ginger with 12 occurrences. The Bendectin theme also remained influential, linking to vomiting with 20 shared occurrences and to double-blind research with 11 occurrences. Additional pathways toward diphenhydramine, birth weight, complementary interventions, and ginger indicate increasing attention to pharmacological treatment, controlled clinical evidence, maternal–fetal outcomes, nutritional support, and non-pharmacological management. Overall, the refined map demonstrates a transition from early drug-exposure and litigation concerns toward a clinically focused obstetric research landscape centered on NVP/HG management, treatment evaluation, supportive care, and pregnancy outcomes.

**Figure 12:**
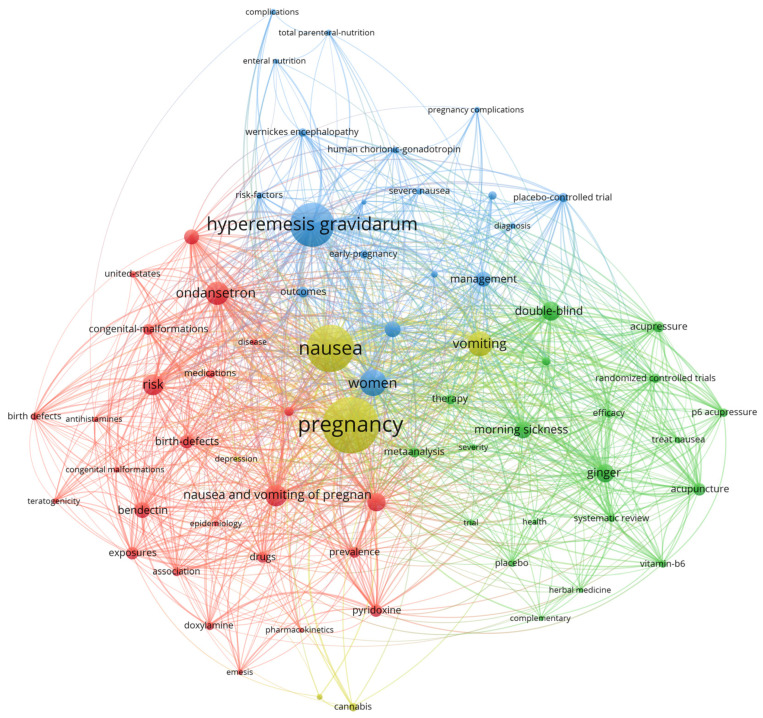
Keyword co-occurrence network of the refined obstetric subset. Author Keywords and Keywords Plus occurring at least ten times were included. Node size is proportional to keyword frequency, line thickness represents co-occurrence strength, and node color indicates the thematic cluster identified by VOSviewer. The map was interpreted as an all-period conceptual structure, with caution due to limited keyword metadata availability before 1991.

The keyword co-occurrence analysis of the refined obstetric subset identified 70 keywords meeting the minimum threshold of ten occurrences, organized by VOSviewer into four clusters (Figure 12). The network was dominated by pregnancy, nausea, HG, women, and vomiting, confirming that its principal conceptual axis was centered on pregnancy-associated nausea and vomiting and its severe clinical form. The strongest co-occurrence relationships were observed between nausea and pregnancy, HG and pregnancy, HG and nausea, nausea and vomiting, and pregnancy and vomiting.

The first cluster represented pharmacological treatment, reproductive safety, and exposure-related research. Its principal terms included ondansetron, nausea and vomiting of pregnancy, risk, safety, Bendectin, antiemetics, pyridoxine, doxylamine, exposure, birth defects, congenital malformations, and teratogenicity. This cluster reflects the longstanding emphasis on antiemetic effectiveness and maternal–fetal safety, particularly regarding medication exposure during early pregnancy.

The second cluster was centered on complementary interventions and controlled treatment evaluation. Prominent terms included double-blind, ginger, morning sickness, acupuncture, acupressure, P6 acupressure, randomized controlled trials, efficacy, vitamin B6, meta-analysis, and systematic review. This grouping indicates a substantial body of research evaluating both non-pharmacological interventions and the quality of evidence supporting their use.

The third cluster reflected the clinical management and complications of HG. HG was its dominant term and was associated with women, metoclopramide, management, outcomes, placebo-controlled trials, corticosteroids, Wernicke encephalopathy, human chorionic gonadotropin, enteral nutrition, total parenteral nutrition, diagnosis, and pregnancy complications. This cluster captures the management of severe disease, including pharmacological therapy, nutritional support, diagnostic assessment, and complication prevention.

The fourth and most centrally positioned cluster was organized around pregnancy, nausea, and vomiting, representing the broad clinical symptom framework connecting the other thematic domains. Overall, the network demonstrates an integrated obstetric research structure linking the clinical spectrum of NVP/HG with medication safety, therapeutic effectiveness, complementary interventions, and supportive management.

### 3.8. Author Co-Citation Network Map

**Figure 13:**
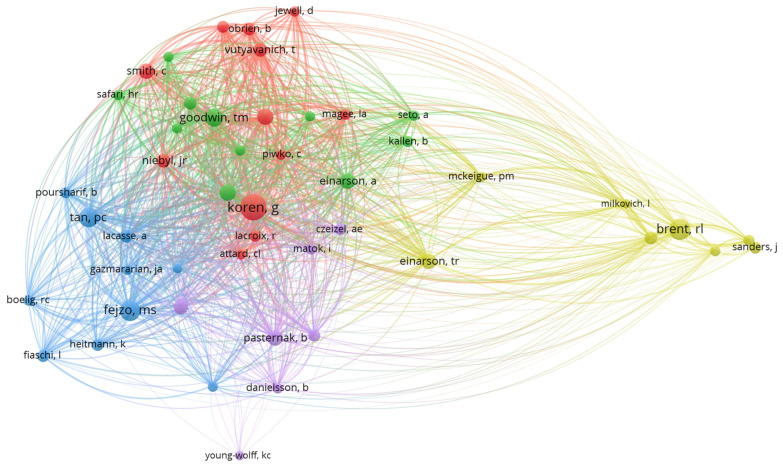
Author co-citation network of the refined NVP/HG obstetric literature. The analysis was based on the 709-record refined subset and included cited authors with at least 40 citations. All 48 authors meeting the threshold formed a single interconnected network divided into five clusters by VOSviewer. Node size is proportional to citation frequency, link thickness represents co-citation strength, spatial proximity indicates greater relatedness, and node color identifies cluster membership.

Author co-citation analysis was conducted using the refined 709-record obstetric subset. A minimum threshold of 40 citations per cited author was applied, yielding 48 cited authors, all of whom formed a single interconnected network comprising 1003 co-citation links (Figure 13). VOSviewer partitioned the network into five clusters containing 12, 11, 10, 8, and 7 authors, respectively. Koren was the most frequently cited and structurally central author, with 295 citations and a total link strength of 3426. Other highly cited authors included Brent (182 citations), Fejzo (174), Tan (156), Goodwin (137), Mazzotta (114), Gadsby (107), Einarson A. (103), Smith (101), Pasternak (96), and Einarson T.R. (95). Fejzo, Tan, Goodwin, Mazzotta, Gadsby, Gill, Smith, Einarson A., Lacasse, and Pasternak also showed high total link strength, indicating prominent bridging positions within the cited-author network.

The first and largest cluster contained 12 authors and was centered on Koren, Mazzotta, Smith, Vutyavanich, and Niebyl. This cluster reflected a clinical-management and intervention-evaluation axis incorporating pharmacological treatment, medication safety, and complementary approaches. The second cluster comprised 11 authors, led by Goodwin, Gadsby, Einarson A., and Gill, and represented clinical epidemiology, disease burden, maternal outcomes, and reproductive medication-safety research. The third cluster contained 10 authors and was organized around Fejzo, Tan, Lacasse, Fiaschi, and Boelig, indicating an HG-focused domain involving disease severity, emerging pathophysiology, population burden, and clinical outcomes. The fourth cluster included eight authors, with Brent, Einarson T.R., Mitchell, Hendrickx, and McKeigue as prominent members, and reflected foundational teratology, congenital-malformation risk, and reproductive-safety evidence. The fifth cluster contained seven authors, including Pasternak, Matthews, Matok, Czeizel, and Danielsson, and represented contemporary pharmacoepidemiology, evidence synthesis, and fetal-safety evaluation of medication or other maternal exposures.

The strongest pairwise co-citation relationship was observed between Fejzo and Tan, with a link strength of 210, followed by Fejzo and Koren (172), Gill and Koren (166), Koren and Mazzotta (161), and Koren and Smith (153). The extensive connections between clusters indicate that the intellectual structure of NVP/HG research is highly interconnected rather than divided into isolated schools of thought. These relationships represent patterns of joint citation and intellectual proximity; they should not be interpreted as direct collaboration, agreement between the cited authors, or evidence that specific researchers independently shaped clinical guidelines.

### 3.9. Cross-Database Coverage Cross-Check

To contextualize the magnitude of potential coverage gaps arising from the single-database design, a supplementary cross-check was performed by re-running an equivalent query in PubMed and Scopus under matched eligibility criteria (English-language articles and reviews published from 1975 to 2025). This comparison was intended as an approximate order-of-magnitude reference rather than a deduplicated coverage analysis; because the three platforms differ in field architecture, phrase indexing, and proximity handling, the query could not be reproduced identically across databases, and the resulting totals reflect gross retrieval rather than counts of uniquely relevant documents. Under these matched criteria, the Web of Science Core Collection corpus comprised 1182 documents, PubMed returned 1227 records, and Scopus returned 2044 records.

The close correspondence between the Web of Science and PubMed totals is consistent with adequate representation of the core biomedical literature on NVP and HG therapy within the Web of Science Core Collection, in that a substantial systematic omission would be expected to produce a markedly lower Web of Science count relative to PubMed. As these totals were not deduplicated, this correspondence does not establish record-level equivalence between the two sets.

The larger Scopus total is consistent with its broader journal indexing base relative to Web of Science [29]. Because the retrievals were not merged or matched at the record level, these totals reflect gross retrieval rather than mutually exclusive document sets. The two databases share a large portion of their biomedical journal coverage, so a substantial fraction of the Scopus records is expected to correspond to publications already contained in the Web of Science corpus. In addition, the intentionally broad Boolean strategy retrieves adjacent literature outside the NVP and HG scope, an effect expected to be more pronounced in Scopus owing to its wider field indexing and looser phrase matching. The gross difference should therefore not be interpreted as the number of uniquely indexed relevant publications absent from the analytical corpus, which is expected to be considerably smaller.

## 4. Discussion

This bibliometric analysis provides a longitudinal overview of research on therapeutic and supportive interventions for NVP and HG across five decades of Web of Science-indexed literature. The findings indicate that the field evolved from a small and irregular body of early publications into a sustained and increasingly diversified research domain. Early work was largely shaped by pregnancy-associated vomiting, drug exposure, and Bendectin-related safety concerns, whereas the contemporary literature is more strongly characterized by pharmacological management, maternal–fetal safety, complementary interventions, diagnostic framing, nutritional and supportive care, maternal–fetal safety, and the evaluation of pharmacological and complementary interventions. This transition reflects not only quantitative growth in publication volume but also a broader conceptual maturation of the field.

To reduce dependence on arbitrarily predefined decades, PELT was used to examine changes in annual publication activity across a range of penalty settings. At full annual resolution, breakpoint locations were distributed across broader transition windows in the mid-1990s and late-2000s/early-2010s rather than converging on the previously reported exact years of 1995 and 2010. The most consistent exact-year signal was observed in 2013, which was detected under six of ten penalties for each tested minimum segment size, while the higher-penalty specifications localized the principal recent transition to 2011. Collectively, these results indicate a sustained early-2010s change in publication activity rather than a uniquely determined single-year rupture.

The three retained macroperiods 1975–1994, 1995–2009, and 2010–2025 should therefore be interpreted as broad descriptive intervals informed by the identified transition windows rather than as exact changepoint-defined phases. Their publication profiles nevertheless demonstrate a clear progression from sparse and irregular early output to intermediate consolidation and, subsequently, marked expansion during the most recent period. The annual-resolution sensitivity analysis consistently localized the principal recent transition to the 2011–2013 interval, with 2013 detected under six of ten penalty settings for each tested minimum segment size.

This transition coincided with an important regulatory milestone in NVP pharmacotherapy. In April 2013, the US Food and Drug Administration approved Diclegis, a delayed-release combination of doxylamine succinate and pyridoxine hydrochloride, for the treatment of NVP in women who had not responded adequately to conservative management [30,31]. The approval restored a medication specifically approved by the FDA for NVP approximately 30 years after the voluntary withdrawal of Bendectin from the US market in 1983 [30]. This interval is more accurately characterized as a gap in specifically FDA-approved NVP pharmacotherapy than as a complete therapeutic void, because other pharmacological options remained available and the individual components of the combination continued to be used in clinical practice [31].

A pivotal randomized, placebo-controlled trial published in 2010 demonstrated the efficacy of delayed-release doxylamine–pyridoxine for the treatment of NVP [10.1016/j.ajog.2010.07.030]. Additional analyses of the randomized-trial data, published after FDA approval, separately evaluated maternal safety and early treatment efficacy [10.1186/s12884-015-0488-1; 10.1186/s12884-016-1172-9]. The renewed regulatory availability of an NVP-specific product, together with continued evaluation of its efficacy, tolerability, and maternal safety, provides a clinically plausible context for the increased scientific attention observed during the early 2010s. Nevertheless, the temporal correspondence between the regulatory milestone and the bibliometric transition does not demonstrate that FDA approval independently caused the subsequent increase in publication output.

Citation dynamics similarly reflect both the maturation of the scientific literature and changes in the clinical organization of NVP/HG care. The high mean citation impact of older publication cohorts, including those from 1991, 1996, and 2005, partly reflects their longer periods of citation accumulation. By contrast, the 2016 publication cohort represents the most prominent recent impact peak, accumulating 2908 citations and showing the strongest post-2010 mean citation impact, at 64.62 citations per article. These values represent citations subsequently received by documents published in 2016 and should not be interpreted as a citation burst occurring during the 2016 calendar year.

The influence of this cohort coincided with a period of increasing international standardization of NVP/HG assessment and management. ACOG Practice Bulletin No. 153, published in September 2015, reviewed the available evidence regarding the diagnosis and management of NVP and emphasized that failure to treat its early manifestations may increase the likelihood of hospital admission for HG [32]. In June 2016, the first edition of RCOG Green-top Guideline No. 69 provided an integrated clinical framework spanning community, ambulatory daycare, and inpatient settings. The guideline incorporated PUQE-based severity classification, explicit indications for inpatient management, escalation across antiemetic classes, intravenous hydration with normal saline and potassium under electrolyte monitoring, thiamine administration before dextrose or parenteral nutrition, and multidisciplinary consideration of enteral or parenteral nutritional support when medical treatment had failed [7].

The same period also produced a major systematic review of pharmacological and non-pharmacological treatments and their associated comprehensive health technology and economic assessment [8,33]. Collectively, these regulatory, guideline, and evidence-synthesis milestones provide a clinically plausible context for the strong subsequent influence of publications from the mid-2010s. However, because the reported indicator aggregates all documents published in 2016, the cohort-level citation peak cannot be attributed to any single guideline, review, regulatory decision, or clinical event.

The structural landscape of NVP/HG intervention literature is led quantitatively by the United States, which contributed the largest absolute volume of publications, total citations, and cross-national collaboration links. However, reducing the geographic interpretation to a static ranking oversimplifies complex patterns of international influence. While the USA serves as the core quantitative hub, countries such as Italy, Australia, and Germany demonstrate high citation efficiency, yielding elevated average citation rates despite smaller publication footprints.

Furthermore, historical and collaborative data reveal a powerful maternal–child health research axis centered in Canada. The University of Toronto and the Hospital for Sick Children (SickKids) formed the single clearest early-to-mid period institutional paradigm, sustaining recurrent publication peaks well before the broader acceleration of American university systems. This specialized concentration highlights Canada’s role in shaping early teratology and reproductive safety frameworks.

Chronologically, the field has transitioned from an early Western concentration (primarily localized within North America and select European nations) toward broader global participation. This globalization is visible in the recent acceleration of output from China, particularly after 2021. Rather than viewing China as a historically mature leader in this specific domain, its recent trajectory indicates its position as an important emerging contributor, diversifying the international research landscape.

The metadata-completeness sensitivity analysis confirmed an important indexing discontinuity that has direct implications for the interpretation of keyword-based bibliometric maps. Web of Science Topic searches are based on title, abstract, author keywords, and Keywords Plus fields, but Clarivate documentation indicates that Author Keywords and Keywords Plus were incorporated into the database from 1991 onward. Consistent with this, our corpus showed almost complete absence of author keywords and Keywords Plus before 1991, with author keyword coverage of only 2.2% and Keywords Plus coverage of 0.0%, compared with 71.8% and 84.9%, respectively, from 1991 onward. Therefore, keyword co-occurrence maps, thematic evolution maps, and trend-topic analyses may be skewed if pre-1991 records are included without adjustment, because older publications are less likely to contribute keyword-based terms even when they are scientifically relevant. This indexing effect may artificially underrepresent early thematic structures and exaggerate the apparent emergence of terms after 1991. For this reason, keyword-dependent analyses were interpreted primarily for the 1991-and-later subset, while earlier records were considered more reliable for title-based, citation-based, and publication-output analyses.

The thematic evolution analysis reinforces the historical interpretation of the field. During 1975–1994, research was concentrated around pregnancy-associated vomiting, Bendectin exposure, early pregnancy, and Bendectin litigation, reflecting the historical prominence of medication-safety and teratogenic-risk concerns. During 1995–2009, these themes consolidated around pregnancy and Bendectin, while burden, diagnosis, total parenteral nutrition, and related supportive-care concepts became more visible. In the most recent period, 2010–2025, the thematic structure diversified toward vomiting, double-blind research, ginger, diphenhydramine, birth weight, and complementary interventions. The continued linkage of Bendectin with vomiting and double-blind research indicates that the historical safety controversy remained connected to subsequent therapeutic evaluation, whereas the expansion from pregnancy toward controlled clinical research and complementary interventions reflects a broader shift toward evidence-based management and maternal–fetal outcome assessment. Because keyword availability was limited before 1991, the earliest thematic pathways should nevertheless be interpreted cautiously, as they may underrepresent the actual conceptual diversity of the early literature.

The appearance of terms such as “double-blind,” “randomized controlled trials,” and “placebo-controlled trials” indicates the increasing thematic visibility of controlled treatment evaluation but does not quantify the proportion of randomized studies relative to observational research. These metadata-derived terms may also occur in reviews, evidence syntheses, or records referring to previous trials and should not be interpreted as verified study-design classifications.

The refined keyword co-occurrence network was organized around a central clinical axis comprising pregnancy, nausea, HG, women, and vomiting. Four interconnected clusters reflected the principal research domains of the field. The first cluster encompassed pharmacological management, reproductive safety, and medication exposure, linking ondansetron, doxylamine, pyridoxine, Bendectin, and antiemetics with risk, safety, first-trimester exposure, birth defects, congenital malformations, and teratogenicity. The second cluster represented complementary interventions and controlled therapeutic evaluation, combining ginger, acupuncture, acupressure, vitamin B6, and morning sickness with double-blind studies, randomized controlled trials, efficacy, meta-analysis, and systematic review. The third cluster was centered on HG and its clinical management and complications, linking HG with metoclopramide, management, outcomes, corticosteroids, Wernicke encephalopathy, human chorionic gonadotropin, enteral nutrition, total parenteral nutrition, diagnosis, severe nausea, and pregnancy complications. The fourth cluster was structured around pregnancy, nausea, and vomiting and acted as a conceptual bridge between the treatment-, safety-, complementary-intervention-, and HG-management-oriented domains. Lower-frequency terms in this cluster included cannabis, marijuana use, and depression, indicating exposure-related and psychosocial subthemes within the broader pregnancy-centered framework. Collectively, the network shows that NVP/HG intervention research integrates maternal symptom control, reproductive safety, controlled treatment evaluation, complementary management, and supportive care for severe disease.

An important emerging development not yet visible as a dominant theme in the keyword maps is the GDF15–GFRAL pathway. Evidence published online in 2023 demonstrated that fetoplacental GDF15 production and maternal sensitivity to this hormone both contribute substantially to the risk and severity of NVP and HG, providing a biologically grounded framework for understanding these conditions and suggesting potential mechanism-based approaches to their prevention and treatment [5]. Its limited visibility in the present maps should be interpreted in relation to the analytical design: the co-occurrence network aggregates five decades of literature, applies a minimum threshold of ten keyword occurrences, and includes publications only through 2025. Consequently, a major but recent mechanistic advance may remain below the visualization threshold because insufficient time has elapsed for a substantial associated literature and stable keyword structure to develop. The present findings therefore do not demonstrate that GDF15 or GFRAL has already displaced established treatment-related terms. Rather, they identify an emerging mechanistic direction whose bibliometric influence cannot yet be evaluated reliably within a five-decade, frequency-thresholded map.

The clinical relevance of the observed thematic structure is supported by highly cited NVP/HG-focused reviews, guideline documents, and outcome-standardization studies within or closely related to the retrieved dataset. Contemporary clinical syntheses emphasize that NVP is common, that HG represents a severe and potentially morbid form of pregnancy-associated nausea and vomiting, and that management may require escalation from dietary or lifestyle measures to pharmacological therapy, hydration, vitamin supplementation, and nutritional support [4,7,34]. The presence of outcome-related terms in the keyword network is also consistent with international efforts to standardize HG research endpoints, including nausea, vomiting, dehydration, oral intake, medication use, maternal wellbeing, pregnancy complications, congenital anomalies, neonatal morbidity, and offspring outcomes [35]. Together, these clinical, guideline, and outcome-standardization papers provide a clinical explanation for the bibliometric structure observed in the present study: the modern NVP/HG literature has shifted beyond symptom description toward severity assessment, treatment escalation, maternal–fetal safety evaluation, and standardized outcome reporting.

The pharmacological cluster should be interpreted through the persistent treatment–safety axis that characterizes medication use in early pregnancy. Large pharmacoepidemiological studies of metoclopramide and ondansetron illustrate how antiemetic use is evaluated not only by therapeutic need but also by fetal outcomes, congenital malformations, oral clefts, and other safety endpoints [36,37,38]. This safety-centered interpretation is further supported by studies of NVP treatment and selected birth defects, as well as data showing the rapid increase in ondansetron prescribing among pregnant women, which reinforced the need for post-marketing safety evaluation [39,40]. In parallel, the complementary-intervention cluster is consistent with the broader evaluation of acupuncture as a non-pharmacological antiemetic approach, including pregnancy-related nausea [41].

The pharmacological keyword structure should not be interpreted as demonstrating a temporal replacement of antihistamines by ondansetron or corticosteroids, because the co-occurrence network aggregates the complete study period rather than measuring changes in keyword frequency over time. Instead, the distribution of these terms is consistent with the clinical differentiation of treatment according to disease severity and treatment line. H1 antihistamines remain established first-line therapies for NVP and HG [33], whereas the prominence of ondansetron reflects its substantial treatment and maternal–fetal safety literature [37,38]. Current evidence indicates that the small possible increase in the absolute risk of orofacial clefting associated with first-trimester ondansetron exposure should be balanced against the maternal and fetal consequences of poorly controlled HG [38]. Corticosteroids occupy a different clinical position and are generally reserved for refractory disease after standard antiemetic regimens have failed [33,42]. Their placement in the HG-centered cluster alongside terms related to severe nausea, management, nutritional support, and pregnancy complications is consistent with treatment escalation in patients at risk of persistent vomiting, dehydration, weight loss, and nutritional compromise rather than routine substitution for antihistamines. Overall, the network structure is more consistent with severity-based and safety-conscious treatment differentiation than with abandonment of established first-line therapy.

The author co-citation network complements the keyword-based findings by showing that the intellectual structure of NVP/HG research is organized around several closely connected traditions rather than isolated schools of thought. The central position of Koren and the dense links involving Mazzotta, Einarson, Goodwin, Gadsby, and Gill reflect the longstanding influence of clinical management, medication-safety, and maternal-outcome research. A separate but strongly connected HG-focused axis, centered on Fejzo and Tan, highlights the growing importance of disease severity, pathophysiology, population burden, and clinical outcomes. The clusters containing Brent, Mitchell, Czeizel, Pasternak, Matok, and related authors further demonstrate the persistent role of teratology, congenital-malformation risk assessment, and pharmacoepidemiological evaluation in shaping the field. The strong cross-cluster links indicate that therapeutic management, fetal safety, clinical epidemiology, and HG-specific research have developed as interdependent components of the same intellectual domain. These co-citation patterns identify authors who are frequently cited together and should not be interpreted as evidence of direct collaboration, scientific agreement, or individual influence on clinical guidelines.

To quantify the degree to which the corpus is centered on pregnancy, records were screened for explicit pregnancy- and obstetric-related terminology, defined as any of the following stems or phrases: pregnancy, gestation, gravid (including HG), obstetric, antenatal, prenatal, perinatal, puerperal, trimester, morning sickness, emesis gravidarum, the abbreviation NVP, the standalone term hyperemesis, and the phrase “nausea and vomiting of pregnancy.” Such wording was present in the title or abstract of 87.0% of records, rising to 93.8% when author keywords and Keywords Plus were also considered. The screen was limited to title, abstract, and keyword metadata rather than full text, and therefore provides a conservative lower bound on topical relevance. This distribution confirms that the corpus is overwhelmingly centered on the NVP and HG literature, with adjacent nausea domains forming a limited periphery consistent with the sensitive search design.

### Limitations

While this study establishes a robust, data-driven overview of the field, several formal methodological limitations must be acknowledged. First, the final analytical dataset was extracted exclusively from the Web of Science Core Collection. While this single-source approach was methodologically necessary to preserve metadata uniformity, eliminate duplicate formatting anomalies, and maintain compatibility with science-mapping environments, it inherently may have excluded relevant publications indexed uniquely in databases such as Scopus, PubMed, Embase, or non-indexed regional journals. Although the eligibility criteria specified English-language articles and reviews, this restriction excluded no records from the retrieved corpus, since all matching documents were already in English (Section 3.2) its practical effect on the present dataset was therefore null. Any broader under-representation of non-English scholarship would arise not from this criterion but from the language and journal coverage of the underlying database, as considered above. A supplementary same-criteria cross-check against PubMed and Scopus, reported in Section 3.8, indicated that the Web of Science corpus closely matched PubMed retrieval while returning fewer gross records than Scopus, consistent with database differences in journal indexing breadth rather than a systematic omission of core NVP and HG literature. This cross-database comparison is itself subject to methodological constraints. The three platforms differ in field architecture, phrase indexing, and proximity operator syntax, so a fully identical query could not be executed and the strategy was instead translated into database-specific equivalents. The PubMed translation was the most approximate of the three, as its field structure required restricting terms to the title and abstract and did not accommodate every proximity clause or specialized term present in the original strategy. The reported totals were also not merged or matched at the record level, so they capture gross retrieval rather than unique, deduplicated coverage. The comparison therefore conveys the approximate relative magnitude of database coverage rather than an exact quantification of the records missed by the single-source design.

Second, long-range text mining across five decades is inherently subject to historical shifts in database engineering. The near-total absence of Author Keywords and Keywords Plus fields prior to 1991 creates an indexing discontinuity that can artificially suppress early concepts and distort the apparent rate of modern thematic diversification. Additionally, citation-based indicators are deeply influenced by publication age, cumulative exposure windows, and journal visibility; consequently, citation volumes and mean impact metrics represent historical bibliometric signals rather than direct measures of clinical evidence quality or current therapeutic utility. Crucially, while keyword-dependent network maps and thematic evolution flows were executed across the entire study timeline to ensure macro-level completeness, the lack of sufficient keyword metadata pre-1991 requires that long-range text trends be evaluated with appropriate caution rather than read as a completely balanced historical record.

Third, the initial search strategy prioritized retrieval sensitivity and therefore retrieved some literature from adjacent nausea and vomiting domains. To improve clinical specificity, a secondary Topic-field exclusion filter was applied before recalculating the country collaboration, keyword co-occurrence, and thematic evolution analyses. Because the exclusion rules operate on any occurrence within the Topic field, they may have removed some relevant pregnancy-focused records that mentioned oncology, anesthesia, or related terminology contextually, while residual non-obstetric records lacking the specified exclusion terms may remain. The remaining bibliometric performance analyses were based on the original 1182-record corpus and should therefore be interpreted separately from the three analyses based on the refined 709-record subset. In addition, the commercial formulation block may not capture every region-specific trade name.

An additional limitation concerns the temporal resolution and clinical interpretation of the PELT analysis. The predefined minimum segment size of three years was selected to reduce over-segmentation caused by random annual variability and to ensure that detected segments represented sustained bibliometric patterns. However, this specification prevents fluctuations confined to one or two years from forming independent publication regimes and therefore prioritizes robustness of long-term macrotrends over sensitivity to immediate scientific responses to safety alerts, regulatory decisions, or epidemiological events. The parameter-sensitivity analysis reduced this concern, as minimum segment sizes of two and three years produced identical breakpoint sets across all tested penalties, while a minimum size of one year differed only at the lowest penalty by permitting an isolated terminal segment in 2025. Nevertheless, PELT was applied exclusively to annual publication counts and not to prescribing patterns, hospitalization rates, disease incidence, guideline adoption, or regulatory activity. Consequently, the absence of a publication breakpoint in 1983 should not be interpreted as evidence that the Bendectin withdrawal lacked an immediate clinical effect. Similarly, although the recent transition was consistently localized to 2011–2013 and included a robust 2013 signal, its temporal proximity to the FDA approval of delayed-release doxylamine–pyridoxine does not establish a causal relationship. The retained macroperiods should therefore be interpreted as broad analytical intervals describing long-term publication development rather than as exact dates of clinical or regulatory transformation.

Fourth, NVP and HG were analyzed as an integrated pregnancy-associated research domain rather than as separate severity-stratified corpora. Although these conditions differ clinically in morbidity, care setting, and therapeutic intensity, the available bibliographic metadata do not provide a standardized disease-severity variable. Many records discuss NVP and HG together, include mixed clinical populations, or evaluate interventions used across more than one severity level. A classification based solely on titles, abstracts, and keywords would therefore risk duplicate inclusion or arbitrary assignment of records, particularly across five decades of changing diagnostic definitions and reporting practices. The resulting clusters were consequently interpreted according to their clinical context, including a distinct severe-HG management and complication domain, rather than being presented as formally stratified NVP and HG subcorpora. Reliable severity-stratified mapping would require record-level clinical classification and, in many cases, full-text assessment.

The author co-citation analysis is also subject to limitations inherent to cited-reference metadata and threshold-based network construction. Cited authors were identified from the abbreviated names recorded in WoSCC, which may occasionally merge different authors with similar names or separate variants belonging to the same individual. The minimum threshold of 40 citations emphasized established contributors and produced an interpretable connected network, but it excluded less frequently cited and recently emerging authors whose influence may not yet be reflected in cumulative citation counts. In addition, co-citation indicates that authors are cited together; it does not demonstrate direct collaboration, agreement, research quality, causal influence on clinical-practice guidelines, or temporal changes in intellectual leadership.

The study also did not classify publications longitudinally as randomized trials, observational studies, systematic reviews, or other study designs. WoSCC records do not provide a standardized study-design variable, and design-related terms in titles, abstracts, Author Keywords, or Keywords Plus cannot reliably distinguish original trials from reviews or publications referring to previous studies. A valid temporal comparison of randomized and observational evidence would require predefined record-level classification and, for many publications, full-text methodological assessment. Accordingly, the presence of terms such as “double-blind,” “randomized controlled trials,” and “placebo-controlled trials” in the thematic maps should be interpreted as evidence of thematic visibility rather than as a quantitative measure of the relative weight or quality of randomized evidence.

Finally, despite implementing a strict string-matching pipeline backed by multi-investigator manual validation and consensus adjudication, automatic or manual keyword thesaurus normalization cannot fully resolve all semantic ambiguity. Language in scientific publishing is inherently evolutionary and context-dependent, meaning that slight variations in terminology across five decades can create granular classification boundaries that standard text-mining frameworks cannot completely eliminate.

## 5. Conclusions

This study provides a data-driven synthesis of the historical development and structural organization of research on therapeutic and supportive interventions for NVP and HG. Across the 1975–2025 period, the field evolved from a sparse and irregular literature centered mainly on pregnancy-associated vomiting, drug exposure, and Bendectin-related safety concerns into a larger, more sustained, and more multidisciplinary research domain. The PELT-derived segmentation identified 1995 and 2010 as key transition points, with the post-2010 period representing the strongest phase of publication expansion.

The contemporary research landscape is organized around a persistent clinical tension in maternal–fetal medicine: the need to relieve maternal symptoms while maintaining rigorous attention to fetal, teratogenic, and reproductive safety. This balance is reflected in the prominence of pharmacological management, reproductive-safety evaluation, complementary modalities, controlled treatment research, diagnostic assessment, nutritional support, and the management of severe HG and its complications. The geographic and institutional findings show that the field remains strongly influenced by North American and Anglo-European contributors, while more recent activity suggests broader international participation.

Methodologically, the study highlights the importance of using data-driven and sensitivity-aware approaches in long-range bibliometric research. PELT analysis reduced reliance on arbitrary chronological divisions, while keyword normalization and metadata-completeness testing helped identify sources of potential distortion in science-mapping outputs. In particular, the sharp improvement in Author Keywords and Keywords Plus coverage after 1991 indicates that thematic evolution and keyword co-occurrence maps must be interpreted cautiously for early publication periods. Future research should prioritize clearer differentiation between NVP and HG, standardized terminology and outcomes, comparative safety studies of pharmacological treatments, stronger evidence for complementary and supportive interventions, and greater representation from underrepresented geographic regions.

## Figures and Tables

**Table 1:** National research contributions in therapeutic and supportive interventions for nausea and vomiting of pregnancy and hyperemesis gravidarum.

Country	Documents	Citations	Average Citations/Document
USA	519	18,036	34.75
Canada	144	4298	29.85
England	84	3899	46.42
Australia	66	4188	63.45
China	46	2327	50.59
Italy	37	2755	74.46
Israel	33	953	28.88
India	30	658	21.93
Brazil	27	205	7.59
Germany	27	1629	60.33

**Table 2:** Source impact of leading publication venues in NVP/HG therapeutic and supportive intervention research.

Source	h_Index	g_Index	m_Index	Total Citations	Publications	Publication Start
OBSTETRICS AND GYNECOLOGY	24	39	0.511	1971	39	1980
AMERICAN JOURNAL OF OBSTETRICS AND GYNECOLOGY	23	28	0.451	1396	28	1976
REPRODUCTIVE TOXICOLOGY	11	21	0.289	503	21	1989
BMC PREGNANCY AND CHILDBIRTH	9	13	0.643	210	13	2013
AMERICAN FAMILY PHYSICIAN	8	10	0.235	290	10	1993
JOURNAL OF MATERNAL-FETAL & NEONATAL MEDICINE	8	10	0.533	304	10	2012
NEUROGASTROENTEROLOGY AND MOTILITY	8	12	0.533	392	12	2012
TERATOLOGY	8	9	0.163	391	9	1978
AMERICAN JOURNAL OF EMERGENCY MEDICINE	7	11	0.5	208	11	2013
AMERICAN JOURNAL OF PERINATOLOGY	7	9	0.233	402	9	1997

h-index, Hirsch index; g-index, Egghe’s g-index; m-index, m-quotient.

## Data Availability

The original contributions presented in this study are included in the article. Further inquiries can be directed to the corresponding authors.

## Supplementary Materials

The following supporting information can be downloaded at: https://www.mdpi.com/article/10.3390/medsci14030419/s1, Table S1. Database-adapted PubMed and Scopus search strategies used for the cross-database record-count comparison; Table S2. PELT minimum-segment sensitivity analysis of annual publication output across penalty values 1–10 (Panel A. Complete results from all 30 parameter combinations; Panel B. Breakpoint detection frequency across the ten penalty settings).

## Abbreviations

The following abbreviations are used in this manuscript:

NVP	Nausea and vomiting in pregnancy
HG	Hyperemesis gravidarum
WoSCC	Web of Science Core Collection
PELT	Pruned Exact Linear Time
TLS	Total link strength
TS	Topic search field
UT	Web of Science accession number
DOI	Digital object identifier
PY	Publication year
TI	Title field
AB	Abstract field
DE	Author keywords field
ID	Keywords Plus field
NFKC	Normalization Form Compatibility Composition
NFKD	Normalization Form Compatibility Decomposition
HTML	HyperText Markup Language
PC6	Pericardium 6 acupuncture point
P6	Pericardium 6 acupuncture point
GDF15	Growth differentiation factor 15
GFRAL	Glial cell-derived neurotrophic factor family receptor alpha-like
5-HT3	5-hydroxytryptamine type 3
RBF	Radial basis function
USA	United States of America
TC	Times cited
MeanTCperArt	Mean times cited per article
