# Supplementary material for: The Therapeutic Landscape of Nausea and Vomiting of Pregnancy and Hyperemesis Gravidarum: Five Decades of Evolving Treatment and Supportive Care
Source: Med Sci (Basel). 2026 Jul 22;14(3):419. doi: 10.3390/medsci14030419 (PMC13413741; doi:10.3390/medsci14030419)
Supplement: Supplementary file 1 [file medsci-14-00419-s001.zip › medsci-4427577-supplementary.pdf]

Table S1. Database-adapted PubMed and Scopus search strategies used for the cross-database record-count comparison.

Database	Query
PubMed	(("nausea and vomiting of pregnancy"[tiab] OR "nausea and vomiting in pregnancy"[tiab] OR "pregnancy nausea"[tiab] OR "nausea in pregnancy"[tiab] OR "vomiting in pregnancy"[tiab] OR "vomiting of pregnancy"[tiab] OR "emesis in pregnancy"[tiab] OR "morning sickness"[tiab] OR "pregnancy sickness"[tiab] OR "gestational nausea"[tiab] OR "gestational vomiting"[tiab] OR "antenatal nausea"[tiab] OR "antenatal vomiting"[tiab] OR "prenatal nausea"[tiab] OR "prenatal vomiting"[tiab] OR "emesis gravidarum"[tiab] OR "hyperemesis gravidarum"[tiab] OR hyperemesis[tiab] OR "pernicious vomiting"[tiab] OR disgravidi*[tiab] OR "pregnancy nausea"[tiab:~10] OR "pregnancy vomiting"[tiab:~10] OR "pregnancy emesis"[tiab:~10]) AND (pyridoxin*[tiab] OR "vitamin B6"[tiab] OR doxylamin*[tiab] OR dimenhydrinat*[tiab] OR diphenhydramin*[tiab] OR promethazin*[tiab] OR meclizin*[tiab] OR meclozin*[tiab] OR cyclizin*[tiab] OR hydroxyzin*[tiab] OR chlorpheniramin*[tiab] OR ondansetron*[tiab] OR metoclopramid*[tiab] OR domperidon*[tiab] OR prochlorperazin*[tiab] OR droperidol*[tiab] OR "5-HT3 antagonist"[tiab] OR mirtazapin*[tiab] OR olanzapin*[tiab] OR gabapentin*[tiab] OR antiemetic*[tiab] OR "anti-emetic"[tiab] OR "P6 acupoint"[tiab] OR PC6[tiab] OR Neiguan[tiab] OR "Sea-Band"[tiab] OR "enteral nutrition"[tiab] OR "parenteral nutrition"[tiab] OR ginger[tiab] OR zingiber[tiab] OR antihistamin*[tiab] OR corticosteroid*[tiab] OR dexamethason*[tiab] OR methylprednisolon*[tiab] OR acupunctur*[tiab] OR acupressure[tiab] OR cannabi*[tiab] OR pharmacotherap*[tiab] OR "intravenous fluid"[tiab] OR rehydrat*[tiab])) OR ("doxylamine-pyridoxine"[tiab] OR "doxylamine pyridoxine"[tiab] OR Bendectin[tiab] OR Debendox[tiab] OR Diclectin[tiab] OR Diclegis[tiab] OR Bonjesta[tiab] OR Xonvea[tiab] OR Cariban[tiab] OR Nacidol[tiab] OR Navidoxine[tiab] OR Pregnea[tiab] OR Emedrin[tiab]) AND english[la] AND ("1975"[dp] : "2025"[dp]) NOT (editorial[pt] OR letter[pt] OR comment[pt] OR news[pt] OR "published erratum"[pt] OR "retracted publication"[pt] OR "expression of concern"[pt])
Scopus	(TITLE-ABS-KEY (("nausea and vomiting of pregnancy" OR "nausea and vomiting in pregnancy" OR "pregnancy nausea" OR "nausea in pregnancy" OR "vomiting in pregnancy" OR "vomiting of pregnancy" OR "emesis in pregnancy" OR "morning sickness" OR "pregnancy sickness" OR "gestational nausea" OR "gestational vomiting" OR "antenatal nausea" OR "antenatal vomiting" OR "prenatal nausea" OR "prenatal vomiting" OR "emesis gravidarum" OR "hyperemesis gravidarum" OR hyperemesis OR "pernicious vomiting" OR disgravidi* OR (NVP W/5 (pregnan* OR gestation* OR gravid* OR hyperemesis OR nausea OR vomit*)) OR (HG W/3 (pregnan* OR gestation* OR gravid* OR hyperemesis)) OR (pregnan* W/10 (nausea OR vomit* OR emesis OR antiemetic*))) AND (pyridoxin* OR "vitamin B6" OR "vitamin-B6" OR doxylamin* OR dimenhydrinat* OR diphenhydramin* OR promethazin* OR meclizin* OR meclozin* OR cyclizin* OR hydroxyzin* OR chlorpheniramin* OR ondansetron* OR metoclopramid* OR domperidon* OR prochlorperazin* OR droperidol* OR "5-HT3 antagonist" OR "5HT3 antagonist" OR mirtazapin* OR olanzapin* OR gabapentin* OR antiemetic* OR "anti-emetic" OR "P6 acupoint" OR "P6 point" OR PC6 OR "PC-6" OR Neiguan OR "Sea-Band" OR "Sea Band" OR "enteral nutrition" OR "parenteral nutrition" OR ((ginger OR zingiber OR antihistamin* OR corticosteroid* OR dexamethason* OR methylprednisolon* OR acupunctur* OR acupressure OR cannabi* OR pharmacotherap* OR "intravenous fluid" OR rehydrat*) W/5 (nausea OR vomit* OR emesis OR hyperemesis OR "morning sickness" OR NVP OR pregnan* OR gestation* OR gravid*)))) OR TITLE-ABS-KEY ("doxylamine-pyridoxine" OR "doxylamine pyridoxine" OR

	Bendectin OR Debendox OR Diclectin OR Diclegis OR Bonjesta OR Xonvea OR Cariban OR Nacidol OR Navidoxine OR Pregnea OR Emedrin)) AND (DOCTYPE (ar) OR DOCTYPE (re)) AND LANGUAGE (english) AND PUBYEAR < 2026 AND (LIMIT-TO (DOCTYPE , "ar") OR LIMIT-TO (DOCTYPE , "re")) AND (LIMIT-TO (LANGUAGE , "English")) AND PUBYEAR > 1974 AND PUBYEAR < 2026

Table S2. PELT minimum-segment sensitivity analysis of annual publication output across penalty values 1–10.

Panel A. Complete results from all 30 parameter combinations.

Minimum segment size (years)	Penalty	Number of changepoints	Detected breakpoint years
1	1	6	1992, 2009, 2013, 2018, 2021, 2025
1	2	4	1992, 2009, 2013, 2018
1	3	3	1996, 2013, 2018
1	4	2	1996, 2013
1	5	2	1996, 2013
1	6	2	1996, 2013
1	7	1	2011
1	8	1	2011
1	9	1	2011
1	10	1	2011
2	1	5	1992, 2009, 2013, 2018, 2021
2	2	4	1992, 2009, 2013, 2018
2	3	3	1996, 2013, 2018
2	4	2	1996, 2013
2	5	2	1996, 2013
2	6	2	1996, 2013
2	7	1	2011
2	8	1	2011
2	9	1	2011
2	10	1	2011
3	1	5	1992, 2009, 2013, 2018, 2021
3	2	4	1992, 2009, 2013, 2018
3	3	3	1996, 2013, 2018
3	4	2	1996, 2013
3	5	2	1996, 2013
3	6	2	1996, 2013
3	7	1	2011
3	8	1	2011
3	9	1	2011
3	10	1	2011

Note: PELT was applied to 51 consecutive annual publication counts (1975–2025) using the radial basis function cost model and jump = 1. The minimum segment size was varied from one to three years, and the penalty ranged from 1 to 10. Reported breakpoint years indicate the first year of the subsequent segment. The final endpoint automatically returned by the ruptures implementation was excluded from the changepoint count.

Panel B. Breakpoint detection frequency across the ten penalty settings.

Minimum segment size (years)	Breakpoint year	Detection frequency, n/10 penalties
1	1992	2/10
1	1996	4/10
1	2009	2/10
1	2011	4/10

Minimum segment size (years)	Breakpoint year	Detection frequency, n/10 penalties
1	2013	6/10
1	2018	3/10
1	2021	1/10
1	2025	1/10
2	1992	2/10
2	1996	4/10
2	2009	2/10
2	2011	4/10
2	2013	6/10
2	2018	3/10
2	2021	1/10
3	1992	2/10
3	1996	4/10
3	2009	2/10
3	2011	4/10
3	2013	6/10
3	2018	3/10
3	2021	1/10

Note: Minimum segment sizes of two and three years produced identical breakpoint sets under all evaluated penalties. A minimum segment size of one year produced the same results under penalties 2–10 and differed only at penalty 1, where a one-year terminal segment beginning in 2025 was permitted. The year 2013 was detected under six of ten penalty settings for every tested minimum segment size. No breakpoint was detected in 1983 in any of the 30 parameter combinations. Under the predefined exact-year robustness criterion (detection in at least five of ten penalty settings), 2013 was the only robust exact-year breakpoint.
